# The Symbiotic Bacterial Profile of Laboratory-Reared and Field-Caught *Aedes albopictus* Mosquitoes from Greece

**DOI:** 10.3390/microorganisms13071486

**Published:** 2025-06-26

**Authors:** Elias Asimakis, Ioannis Galiatsatos, Georgia Apostolopoulou, Eleni C. Savvidou, Georgios Balatsos, Vasileios Karras, Vasiliki Evangelou, Eva Dionyssopoulou, Antonios Augustinos, Nikos T. Papadopoulos, Antonios Michaelakis, Panagiota Stathopoulou, George Tsiamis

**Affiliations:** 1 Laboratory of Systems Microbiology and Applied Genomics, Department of Sustainable Agriculture, University of Patras, 2 George Seferi St., 30131 Agrinio, Greece; eliasasim@upatras.gr (E.A.); jgalia96@gmail.com (I.G.); gogoapostolopoyloy1@gmail.com (G.A.); edionys@upatras.gr (E.D.); panstath@upatras.gr (P.S.); 2 Laboratory of Entomology and Agricultural Zoology, Department of Agriculture, Crop Production and Rural Environment, University of Thessaly, Fytokou St., 38446 Volos, Greece; eleni241986@gmail.com (E.C.S.); nikopap@uth.gr (N.T.P.); 3 Scientific Directorate of Entomology and Agricultural Zoology, Benaki Phytopathological Institute, 14561 Kifissia, Greece; g.balatsos@bpi.gr (G.B.); v.karras@bpi.gr (V.K.); v.evangelou@bpi.gr (V.E.); a.michaelakis@bpi.gr (A.M.); 4 Department of Plant Protection Patras, Institute of Industrial and Forage Crops, Hellenic Agricultural Organization ‘DIMITRA’, 26442 Patras, Greece; antoniosaugustinos@gmail.com

**Keywords:** asian tiger mosquito, SIT, combined SIT/IIT approach, insect mass-rearing, vector-borne disease, insect microbiome, 16S rRNA amplicon metagenome, artificial diet supplements, insect probiotics

## Abstract

The Asian tiger mosquito *Aedes albopictus* is a highly invasive species capable of transmitting human pathogens. For population management, the sterile insect technique (SIT) is considered an effective and sustainable alternative to conventional methods, such as insecticides and reducing or eliminating breeding sites. The use of symbiotic bacteria to improve the application of SIT or design combined SIT/incompatible insect technique (IIT) approaches is currently considered. In this context, exploring the microbiota of local mosquito populations is crucial for identifying interesting components. This study employed 16S rRNA sequencing and microbiological methods to characterize the diversity of laboratory and wild *Ae. albopictus* in Greece. Differences were recorded between wild and lab-reared mosquitoes, with laboratory samples exhibiting higher diversity. Laboratory treatment, sex, and developmental stage also resulted in variations between communities. Populations reared in the same facility developed mostly similar bacterial profiles. Two geographically distant wild populations displayed similar bacterial profiles, characterized by seasonal changes in the relative abundance of *Pantoea* and *Zymobacter*. *Wolbachia* was dominant in most groups (63.7% relative abundance), especially in field-caught mosquitoes. It was identified with two strains, *w*AlbA (21.5%) and *w*AlbB (42.2%). Other frequent taxa included *Elizabethkingia*, *Asaia*, and *Serratia*. Blood feeding favored an increase in *Serratia* abundance. Various *Enterobacter*, *Klebsiella*, *Aeromonas*, and *Acinetobacter* strains were isolated from larval and adult mosquito extracts and could be further characterized as diet supplements. These findings suggest that the microbiota of local populations is highly variable due to multiple factors. However, they retain core elements shared across populations that may exhibit valuable nutritional or functional roles and could be exploited to improve SIT processes.

## 1. Introduction

*Aedes* (*Stegomyia*) *albopictus* (Skuse) (Diptera: Culicidae) is considered one of the most important mosquito species due to its high vectorial capacity and invasiveness that is facilitated by highly plastic and adaptive responses to new environments. As a competent vector of pathogens, it constantly threatens public health by transmitting severe infectious diseases such as Chikungunya, Dengue, Zika, and yellow fever [1]. Due to the species’ ecology and high invasion rates, constant monitoring and effective management of its populations are crucial [2].

Compared to the classic management methods, such as the reduction/elimination of breeding sites and insecticide application, the Sterile Insect Technique (SIT) is considered one of the most effective and environmentally friendly alternatives [3,4]. This approach is based on sterile male releases in the field which can gradually suppress mosquito populations by inducing sterility in eggs laid by feral females [5,6]. Despite the high efficacy and low environmental impact of SIT, the high cost of mosquito mass rearing impedes its application. Alternative use of insect gut bacteria in mass-rearing procedures appears to be a field of extended research that could potentially support SIT by decreasing costs and sustaining or improving reared insect quality. For example, a larval diet enriched with gut bacteria enhanced the mass-rearing of the fruit fly *Ceratitis capitata* (Wiedemann) (Diptera: Tephritidae). Mass-produced males exhibited increased lifespan and adult size, and improved flight ability and mating performance (Figure S1) [7,8,9,10,11]. Despite previous research on agricultural pests, the advantages of exploiting gut bacteria for mosquito management have not been extensively studied, although studies exist in this field [12,13]. The characterization of the mosquito microbiome is the first step towards identifying potential candidates to implement in SIT processes. Such analyses provide insight into the dynamics of the community, including the presence or absence of members and putative interactions, under various conditions. Subsequent steps usually include isolation and testing of strains for beneficial effects. Various multi-omics approaches are used to reveal genomic and functional properties of candidate strains, including detailed metabolic profiles and putative pathogenicity mechanisms [14].

The microbiota of *Aedes* mosquitoes have been characterized and compared among different species, habitats, populations, sexes, and developmental stages. They are mainly composed of bacteria belonging to the phyla Proteobacteria, Bacteroides, Firmicutes, and Actinobacteria, including representatives of the families Enterobacteriaceae, Erwiniaceae, Yersiniaceae, Acetobacteraceae, Enterococcaceae, and Bacillaceae [14,15,16,17,18]. Similar to other insects, gut symbionts play crucial roles in host physiology, especially development, fecundity, and survival. These roles are mostly fulfilled by contributing to the digestion and synthesis of vital substances, such as carbohydrates, vitamins, and amino acids. Symbiotic bacteria may also affect host fitness and immunity, thereby regulating mosquito vector competence and disease patterns among populations [19,20,21,22]. For example, bacteria located in various tissues, such as *Wolbachia*, *Enterobacter*, and *Asaia*, can interfere with mosquito-borne viruses or induce host immune responses against *Plasmodium*, parasitic filarial nematodes, and pathogenic bacteria (Figure S1) [16,22]. However, the interaction between bacteria and pathogens is not always straightforward, as some strains can enhance the proliferation or transmission of viruses [23,24]. Moreover, the microbial communities themselves are affected by host pathogens, with various taxa increasing or decreasing in response to microbial or parasitic infections [16].

Among the bacterial genera colonizing *Ae*. *albopictus*, *Wolbachia* (Alphaproteobacteria: Rickettsiales) is of particular interest. *Wolbachia* is an integral part of the microbiota of *Ae. albopictus*, often present with a high incidence and prevalence in the studied populations [25]. Its persistence in populations is facilitated by its ability to be transmitted vertically, from parents to offspring, and horizontally across species [26]. Numerous *Wolbachia* strains can induce a series of reproductive phenotypes in their hosts, with cytoplasmic incompatibility (CI) being the most common [26,27]. As a reproductive parasite, *Wolbachia* is primarily associated with reproductive tissues, although infections can be detected throughout the insect body [28,29]. In *Ae*. *albopictus*, *Wolbachia* is generally identified with two strains, *w*AlbA and *w*AlbB, which usually show differential presence among male and female individuals [30]. Modern approaches suggest the use of *Wolbachia*-induced CI for population replacement or population suppression (Figure S1). The first approach uses *Wolbachia*-infected laboratory populations, which exhibit reduced vectorial capacity owing to the presence of the bacterium, to replace infectious wild populations. In the second case, the incompatible insect technique (IIT) which is analogous to SIT, can directly suppress the vector population. The final outcome of both approaches is the suppression of vector-borne diseases [31,32,33,34]. In this context, both SIT and *Wolbachia*-based IIT approaches have been successfully tested [35,36]. In the absence of a perfect sexing strategy that would entirely eliminate female contamination in male release batches, the combined SIT/IIT has been proposed as a viable alternative to avoid releasing fertile females that carry *Wolbachia* strains not present in the natural population [36,37,38]. In this approach, sterility is delivered to a natural population through laboratory males carrying incompatible *Wolbachia* strains. The transinfected males retain high competitiveness due to the presence of *Wolbachia* and avoid the fitness costs associated with irradiation treatment. At the same time, low irradiation doses ensure that no fertile females are released in the field. High-throughput sequencing has improved the monitoring of *Wolbachia* incidence and prevalence in wild populations, which is crucial for identifying incompatible strains and designing IIT-based approaches.

A better understanding of the mosquito gut microbiota and their possible uses seems crucial to support control strategies, such as SIT, IIT, or the combined SIT/IIT approach [37]. Since most studies on the Greek population of *Ae*. *albopictus* focus primarily on *Wolbachia* and *Asaia* [39,40], the overall composition of the bacterial communities remains largely unexplored. Therefore, we sought to analyze the bacterial profiles of field-caught, and laboratory-reared populations of *Ae*. *albopictus* collected from various areas of the country. We examined how geographic origin and treatment under laboratory conditions affect the structure and composition of bacterial communities. Such knowledge could be crucial when selecting a wild population for laboratory domestication or when designing a containment strategy against a local population based on SIT or combined SIT/IIT approaches. Since larvae and adult mosquitoes are characterized by different dietary habits and nutritional needs [14,41], it is interesting to examine whether these developmental stages share common or contain unique bacterial consortia. In both cases, bacterial strains may be isolated, cultivated, and tested as food supplements to support artificial rearing at each life stage. To address these questions, we analyzed the bacterial diversity within six laboratory populations of *Ae*. *albopictus*, originating from geographically distinct regions of Greece. These populations were maintained in two separate laboratory facilities. Additionally, we examined two natural populations of *Ae*. *albopictus*. Our metagenomic amplicon analysis utilized Illumina MiSeq sequencing of the 16S rRNA gene. Factors such as geographic origin, laboratory rearing conditions, developmental stage, and mosquito sex were examined. Furthermore, we explored the culturable bacterial diversity in mosquito larvae and adults to isolate beneficial strains that could enhance laboratory or mass-rearing procedures for *Ae*. *albopictus*.

## 2. Materials and Methods

### 2.1. Housing and Rearing Conditions for Laboratory Populations

**Figure 1:**
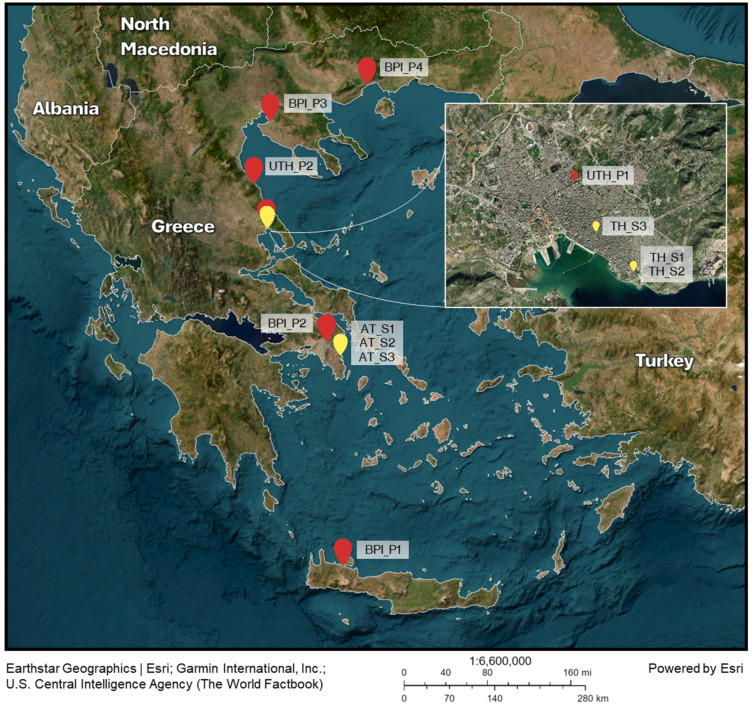
Sampling sites for eggs (red pins) and wild adult mosquitoes (yellow pins). Collected eggs were used to develop the laboratory populations in BPI and UTH (image created with ArcGIS Online by Esri).

The laboratory populations were maintained at the Laboratory of Entomology and Agricultural Zoology, University of Thessaly (UTH), and the Laboratory of Insects and Parasites of Medical Importance, Benaki Phytopathological Institute (BPI). Six populations were analyzed, four from the BPI and two from the UTH. The populations originated from eggs collected with a network of ovitraps installed in various areas of Greece, including Chania (BPI_P1), Attica (BPI_P2), Thessaloniki (BPI_P3), Kavala (BPI_P4), Volos (UTH_P1), and Karitsa (UTH_P2) (Figure 1, Table S1). The network is used for the continuous surveillance of *Ae*. *albopictus* populations in the country. Moreover, these areas account for 71.7% of the country’s population (according to the latest national consensus of 2021). The four populations from BPI were developed from newly collected eggs laid on wooden substrates attached to water-filled ovitraps [42,43]. Egg hatching was carried out in 1 L beakers containing 700 mL of water and 2 mL of a deionized water solution consisting of 12.5% *w*/*v* nutrient broth powder (Oxoid, Basingstoke, UK) and 2.5% *w*/*v* yeast extract powder (Oxoid, Basingstoke, UK) [44]. First, instar larvae were placed in large plastic containers at a density of 2 larvae/mL, supplied with a daily provision of fish food (0.1 mg/larva/day) (“NovoTom Artemia”, JBL, Neuhofen, Germany) until the pupal stage [45,46,47,48]. Developed pupae were recorded and collected daily. They were subsequently placed in 30 × 30 × 30 or 15 × 15 × 15 cm plexiglass cages with constant access to a 10% *w*/*v* sugar solution, where they developed into adult mosquitoes. Bovine blood meals were offered to sample groups of female mosquitoes from the BPI lab (blood-fed, BF groups) for 30 min every four days, using a Hemotek membrane feeding system (Hemotek Ltd., Blackburn, UK) [49]. Rearing was performed under controlled temperature (25 ± 2 °C), relative humidity (65 ± 5%), and lighting conditions (L14:D10, with sunset and sunrise simulations for 30 min).

The two UTH populations, UTH_P1, and UTH_P2 originated from eggs collected with ovitraps installed in the areas of Volos and Karitsa and have been reared under laboratory conditions since 2017 (>F_20_) and 2021 (F_5_), respectively. Housing and rearing procedures for these populations have been previously described in detail [50], with larval density estimated at 0.75 larvae/mL. Before sampling, rearing conditions for the juvenile and adult stages were adjusted to match the processes described above for the BPI populations. The average time between laboratory generations ranged from 35 to 40 days, including the period from eggs to sexually mature adults. Adults typically lived for 1 to 3 months.

### 2.2. Sample Collection and Preparation from Laboratory Populations

Sampling for the 16S rRNA amplicon survey of bacterial communities involved third and fourth instar larvae and 1-day, 3-day, and 14-day-old male and female adults. Different age groups for males, females, and larvae were merged, as age had no effect on their bacterial communities (*p* > 0.05). Additionally, for the four BPI lab populations, 14-day-old blood-fed (BF) and non-blood-fed (NBF) female mosquitoes were also collected (Table 1) at a different period from the previous samples. Each sample consisted of five individuals pooled in 1.5 mL Eppendorf tubes containing 100 μL of RNA stabilizing reagent (fix RNA, EURX, Gdańsk, Poland). Samples were stored at 4 °C until further processing. Larvae were pooled immediately into 1.5 mL Eppendorf tubes. Adults were collected from plexiglass cages using custom mechanical aspirators, transferred into 50 mL falcon tubes, and placed at −20 °C for 5 min. After treatment, mosquitoes were transferred into 1.5 mL Eppendorf tubes using sterile forceps.

### 2.3. Sample Collection and Preparation from Wild Populations

Wild individuals were collected using the human landing catch method [51]. Mosquito samples (adults) were collected from two areas: Vravrona, in the region of Attica (AT), and Volos, in the region of Thessaly (TH) (Figure 1). Sites in Vravrona have been used for pilot SIT field tests against *Ae*. *albopictus* [5,52]. Three expeditions were performed in June, August, and October 2022. Both female and male adults were collected by experienced entomologists using custom-made mechanical aspirators equipped with a fan connected to a 12-volt car battery. Captured mosquitoes were maintained at 4 °C for 12 h and then placed at −20 °C for 5 min. Species identification was performed using a stereoscope and dichotomous keys [53]. Mosquitoes that were successfully identified as *Ae. albopictus* were individually placed in 1.5 mL Eppendorf tubes containing 100 μL of RNA stabilizing reagent (*fix* RNA, EURX, Poland). Twenty mosquitoes were collected from each area during each expedition, resulting in a total of 120 adults (Table S2).

Additional larval and adult samples were collected during August in Vravrona, Attica (AT) for the culture-dependent approach. Approximately 100 adult mosquitoes were collected and pooled in a 50 mL falcon tube with RNA stabilizing reagent (*fix* RNA, EURX, Poland). Moreover, 500 mL of water containing ~100 *Ae. albopictus* larvae were collected in a 1 L bottle. All samples were stored at 4 °C before they were used for the preparation of the homogenates.

### 2.4. Culture Preparation and Molecular Identification of Isolates

Separate homogenates were prepared from wild adult mosquitoes and larvae captured in Attica (AT) during an expedition in August 2022. Initially, the samples were surface sterilized with a 70% *v*/*v* ethanol solution and washed with sterile deionized water. Then, 50 individuals from each life stage were pooled in separate 50 mL falcon tubes, homogenized in 5 mL of sterile 1X phosphate-buffered saline (PBS) solution (137 mM NaCl, 2.7 mM KCl, 4.3 mM Na_2_HPO_4_, and 1.47 mM KH_2_PO_4,_ pH 7.4) and vortexed for 2 h. Ten-fold serial dilutions (10^−1^ to 10^−6^) were prepared from the two homogenates in 900 μL of sterile 1X PBS solution. Additionally, enriched homogenates were prepared by diluting 500 μL of each homogenate with 1 mL of tryptone soy broth (Neogen, Heywood, UK). Ten-fold serial dilutions (10^−1^ to 10^−6^) were also prepared for the enriched homogenates in 900 μL of sterile tryptone soy broth. Subsequently, 100 μL of the initial and all serially diluted homogenates were plated on three nutrient media: LB Agar (1% *w*/*v* peptone, 1% *w*/*v* NaCl, 0.5% *w*/*v* yeast extract and 1.5% *w*/*v* agar), PDA (Neogen, Heywood, UK), and MacConkey Agar (Neogen, Heywood, UK). Plates were incubated at room temperature (25 °C) for up to 48 h and stored at 4 °C when visible growth was observed. Morphologically different colonies (shape, color, etc.) were individually selected and re-streaked at least three times on the appropriate solid medium to obtain pure cultures.

Colony identification was based on amplification and Sanger sequencing of the bacterial 16S rRNA gene with 27F/1492R primers (~1465 bp fragment) [54]. Single colonies were picked from pure cultures and used directly as templates for colony PCR [55]. Amplification was performed in 25 μL reactions with the KAPA Taq PCR Kit (Roche, Basel, Switzerland). Each reaction contained 1X KAPA Taq buffer A (1.5 mM of MgCl_2_), 0.2 mM from each dNTP solution, 0.4 μM from each primer solution, 0.5 U of KAPA Taq DNA polymerase, a single colony as template, and sterile deionized water. The cycling protocol involved an initial denaturation step at 95 °C for 10 min, followed by 35 cycles of 95 °C for 30 s, 53 °C for 30 s, and 72 °C for 2 min, and a final extension step at 72 °C for 5 min.

### 2.5. DNA Extraction

Initially, larvae and adult *Ae. albopictus* were surface sterilized using a 70% *v*/*v* ethanol solution and then washed with sterile deionized water. DNA was extracted from each sample using 60 μL of cetyltrimethylammonium bromide (CTAB) extraction buffer (2% CTAB, 100 mM Tris-HCl pH 8, 20 mM EDTA pH 8, 1.4 M NaCl) and 0.6 μL 2-mercaptoethanol (GIBCO, New York, NY, USA). After homogenization, samples were incubated at 65 °C for 5 h. An equal volume of chloroform/isoamyl alcohol (24:1) solution was used for extraction. Samples were centrifuged at 14,000× *g* for 15 min at room temperature for phase separation. The supernatant aqueous phase was transferred to a microcentrifuge tube. DNA was precipitated by adding 60 μL isopropanol, incubated at −20 °C overnight, and centrifuged at 14,000× *g* for 20 min at 4 °C. The precipitate was washed with 180 μL 70% *v*/*v* ethanol solution and centrifuged for 10 min at 14,000× *g* at 4 °C. The supernatant was discarded, and the precipitate was left to dry for 10–15 min at 37 °C. The pellet was resuspended in 60 µL of sterile deionized water. A Q5000 microvolume UV-vis spectrophotometer (Quawell Technology, San Jose, CA, USA) was used to assess the quality and quantity of the extracted DNA samples. After quantitation, DNA samples were preserved at −20 °C.

### 2.6. PCR Amplification of the 16S rRNA and COI Genes

PCR amplification was performed in 25 μL reactions using the KAPA Taq PCR Kit (Roche, Basel, Switzerland). Individual reactions were prepared by mixing 1X KAPA Taq buffer A (contains 1.5 mM of MgCl_2_), an additional 1 mM of MgCl_2_, 0.2 mM from each dNTP solution, 0.4 μM from each primer solution, 0.5 U of KAPA Taq DNA Polymerase, ≤250 ng of template DNA, and the required volume of sterile deionized water to finalize the reaction. The amplification protocol for the 16S rRNA gene (for Illumina sequencing) included an initial denaturation step at 95 °C for 5 min, 40 cycles at 95 °C for 30 s, 50 °C for 30 s, and 72 °C for 30 s, followed by a final extension step at 72 °C for 5 min. A 344 bp fragment that included the V4-V5 variable regions of the 16S rRNA gene was the target of the amplification, using the 564F (5′-AYTGGGYDTAAAGNG-3′) and 908R (5′-GCGTCAATTCMTTTGAGIT-3′) primer set [56]. Species verification for wild mosquitoes, which were initially identified during sample collection using dichotomous keys, was performed by PCR amplification of an ~800 bp fragment of the mitochondrial gene coding for cytochrome c oxidase subunit I (COI). The fragments were amplified using species-specific primers for *Ae. albopictus* 2027F (5′-CCCGTATTAGCCGGAGCTAT-3′) and 2886R (5′-ATGGGGAAAGAAGGAGTTCG-3′) [57]. The cycling protocol included initial denaturation at 95 °C for 5 min, followed by 35 cycles at 95 °C for 30 s, 55 °C for 30 s, and 72 °C for 1 min. The reaction was finalized with an extension step at 72 °C for 2 min.

### 2.7. Evaluation of PCR Results, Purification of Positive Reactions, and Sanger Sequencing

PCR amplicons were visualized on a Gel Doc XR+ system (Biorad, Hercules, CA, USA) after separation with 1.5% *w*/*v* agarose gel electrophoresis in 1X TAE buffer (40 mM Tris-acetate, 1 mM EDTA). Positive PCR products were purified using 20% *w*/*v* polyethylene glycol (PEG)/2.5 M NaCl solution. The reactions were mixed with an equal volume of solution and centrifuged at 14,000× *g* for 20 min. The precipitate was rinsed with 125 μL of 70% *v*/*v* ethanol and centrifuged at 14,000× *g* for 10 min. After performing the above wash step twice, the precipitate was left to dry at 37 °C for 10–15 min and resuspended in 15 μL of sterile deionized water. Purified 27F/1492R amplicons were used to prepare chain termination (Sanger) sequencing reactions with the ABI BigDye Terminator v3.1 Cycle Sequencing kit, according to the manufacturer’s recommendations (Applied Biosystems, Foster City, CA, USA). Two sequencing reactions were performed for each purified PCR product using either 27F or 1492R in each reaction. Reactions were purified with an ethanol/EDTA protocol following the manufacturer’s instructions (Applied Biosystems, Foster City, CA, USA). Sequencing was performed using an ABI 3730xl DNA Analyzer (Applied Biosystems, Foster City, CA, USA).

### 2.8. Illumina Library Preparation and High-Throughput Sequencing

The Illumina indexes were attached to the purified 16S rRNA amplicons with a second PCR. Each sample was amplified with a unique combination of forward and reverse index primers. Amplification was carried out in 50 μL reactions using the KAPA Taq PCR Kit (Roche, Basel, Switzerland). Each reaction contained 1X KAPA Taq buffer A (1.5 mM of MgCl_2_), 0.2 mM from each dNTP solution, 1 μM from each index primer, 0.5 U of KAPA Taq DNA Polymerase, 20 ng of template DNA, and sterile deionized water. The cycling protocol included initial denaturation at 95 °C for 5 min, 8 cycles of 95 °C for 30 s, 54 °C for 30 s, and 72 °C for 45 s, and a final extension step at 72 °C for 5 min. Prior to sequencing, the reactions were purified with magnetic beads using the NucleoMag^®^ NGS Clean-up and Size Selection kit, following the manufacturer’s instructions (Macherey-Nagel, Düren, Germany). The samples were eluted with 30 μL of sterile deionized water, quantified, diluted to equimolar volumes, and pooled to create the final sequencing library. Finally, 2 × 250 bp paired-end sequencing was performed by Macrogen Europe BV (Amsterdam, The Netherlands) on the Illumina MiSeq Sequencing System.

### 2.9. Data Analysis

Raw sequencing reads were processed using USEARCH v11 [58]. Initially, paired-end reads were assembled using the -fastq_mergepairs command. Short and long merged sequences were also discarded during this step. The -fastq_filter command was used to discard reads with at least one expected error (E_max_ = 1) in their sequences. Unique reads and their frequencies were detected with the -fastx_uniques command. Sequences were clustered into operational taxonomic units (OTUs) with at least 97% sequence similarity using the UPARSE-OTU algorithm [59] implemented in the -cluster_otus command. The algorithm also removes singleton and chimeric sequences. The -otu_tab command was used to create the OTU table, which contains the number of reads per sample and OTU. Incorrectly assigned reads were identified and filtered using the UNCROSS2 algorithm [60] with the -otutab_xtalk command. Low-abundance OTUs (minimum abundance threshold 0.1%) were removed from the table with—otutab_trim. The resulting OTUs were assigned taxonomy with QIIME 2 [61] using BLAST+ v2.16.0 [62] and SILVA database, release 138 [63]. Raw data from this study were deposited into the NCBI SRA archive under the accession number PRJNA1177906.

Alpha and beta diversity, relative abundance, core microbiome, and statistical analyses of Illumina data were performed using MetaXplore v1.0 [64]. The composition of bacterial communities was assessed with beta diversity analysis using the weighted UniFrac distance and visualized using Canonical Analysis of Principal coordinates (CAP) plots. Permutational multivariate analysis of variance (PERMANOVA) using distance matrices was used to identify significant differences between groups. A *p*-value of ≤0.05 was considered indicative of statistical significance. The homogeneity of group dispersions was calculated using the betadisper function in the R package vegan v2.7-1 [65]. ANOVA tests were used to test for overall differences in dispersion and Tukey’s Honest Significant Difference (HSD) for pairwise comparisons. A *p*-value of ≤0.05 indicated statistically significant differences in dispersion between groups. Only statistically different dispersions are presented in the text. The core microbiome was calculated using a 75% prevalence threshold (number of samples in which an OTU was present) and a 0.01% relative abundance threshold per OTU. Relative abundance heatmaps, Venn diagrams, and CAP plots were generated using MetaXplore [64]. The sampling point map was generated using ArcGIS ^®^ Online by Esri (www.esri.com).

Chromatograms derived from Sanger sequencing were processed using Geneious v7.0.6 [66]. Processing included trimming low-quality regions, assembly of sequencing fragments, and manual correction of ambiguities to produce the final consensus sequence. Processed sequences were submitted to BLASTn v2.16.0 [67] searches against the default nr/nt database and GenBank’s rRNA/ITS database [68]. Phylogenetic analysis of 16S rRNA sequences from *Wolbachia* OTUs (OTU1 and OTU3) and bacterial isolates was performed using Geneious v7.0.6. Multiple alignments were performed with MUSCLE v5.3 [69]. Phylogenetic relationships were visualized using neighbor-joining trees based on the Tamura-Nei genetic distance model [70]. Trees were resampled 1000 times using the bootstrap method. Outgroup sequences were used for *Wolbachia* and bacterial isolates 16S rRNA trees. The derived 16S rRNA sequences were deposited into GenBank under the accession numbers PQ505081-PQ505091 and PQ505106-PQ505107.

## 3. Results

### 3.1. Dataset Information and Taxonomic Assignment of Filtered Reads

The initial sequencing dataset consisted of 10,654,199 raw reads with 3.84 Gbps total length and 29,350 reads on average per sample. After quality control and trimming, 2,884,486 high-quality reads with a total length of approximately 1 Gbps remained in the dataset and were used in the analysis. This resulted in 7946 filtered reads per sample. In total, 363 samples were included in the 16S rRNA amplicon sequencing analysis of bacterial communities. These consisted of 243 and 120 samples from the laboratory and wild populations of *Ae. albopictus*, respectively.

Filtered reads were clustered into forty-three OTUs. Forty bacterial OTUs were identified in laboratory-reared samples and thirty-seven in wild samples. Therefore, six OTUs (OTU6, OTU8, OTU15, OTU16, OTU35, and OTU38) were unique to the laboratory samples, and three OTUs (OTU7, OTU25, and OTU27) were found only in wild *Ae. albopictus* samples. The remaining 34 OTUs were shared between the two types of samples. The 43 OTUs were grouped into thirty-four bacterial genera, twenty-six families, twenty orders, five classes, and four phyla (Table S3). One OTU (OTU106) remained unassigned at the 97% similarity threshold. This group showed 90.53% similarity with the genus *Dysgonomonas* and could be considered a putative new genus in the family Dysgonomonadaceae. Proteobacteria was the prevalent phylum (average relative abundance ± SE: 81.4 ± 1.4%), followed by Bacteroidota (12 ± 1.1%), Actinobacteriota (5.91 ± 0.9%), and Firmicutes (0.67 ± 0.2%). Despite being the prevalent phylum, Proteobacteria showed a varied distribution in the studied populations, from 26.8 to 99.6% (Figure S2). Similar variance was also observed in the other three phyla, Bacteroidota (0.2–72.4%), Actinobacteriota (0–18.2%), and to a lesser degree, Firmicutes (0–3.1%). The alphaproteobacterium *Wolbachia* was dominant, accounting for 63.7% of the reads and was present in the dataset with two OTUs (Table S3).

Despite the large number of shared OTUs between the laboratory and wild populations, statistical analysis revealed significant differences in the structure of their bacterial communities (PERMANOVA, *p* ≤ 0.05; betadisper, *p* ≤ 0.05). Laboratory samples contained richer and more diverse bacterial communities than wild samples (*p* ≤ 0.05) (Table S4). *Wolbachia* was once again dominant in both sample types. It was present with a 53.1% relative abundance in laboratory samples and 85.3% in wild mosquitoes (Figure S3). Laboratory-reared mosquitoes were also characterized by a strong presence of *Elizabethkingia* (16.4%), *Microbacterium* (7.7%), and *Asaia* (5.9%), while wild samples by *Zymobacter* (5.6%), and *Pantoea* (4.4%).

### 3.2. The Bacterial Communities of the Laboratory Populations

**Figure 2:**
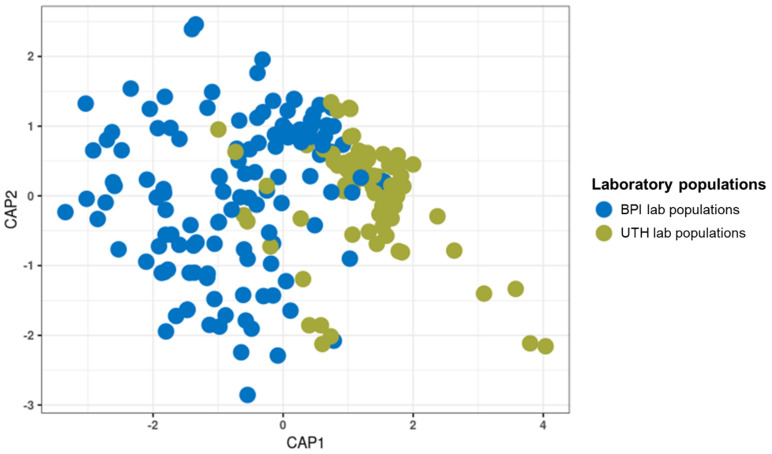
Canonical analysis of principal coordinates for the bacterial communities of laboratory samples from the BPI and the UTH. The samples formed two distinct clusters due to significant differences (PERMANOVA, *p* = 0.001; betadisper, *p* ≤ 0.05).

The structure of the bacterial communities differed between the laboratory populations of the BPI (Attica) and UTH (Thessaly) (PERMANOVA, *p* ≤ 0.05; betadisper, *p* ≤ 0.05). These differences resulted in the formation of two separate clusters on the CAP plot for the two groups (Figure 2). The analysis included 204 samples, 124 samples from four laboratory populations of the BPI and 80 samples from two laboratory populations of the UTH (Table 1). Thirty-nine blood-fed mosquitoes and their controls from the BPI were excluded from the comparison because they had no corresponding samples in the UTH populations. *Wolbachia* was, as expected, the prevalent bacterial genus (46% in BPI and 75.2% in UTH) (Figure S4). BPI populations were also characterized by the presence of *Elizabethkingia* (16.6%), *Microbacterium* (14.7%), *Asaia* (5.7%), and *Serratia* (2.5%). In the UTH samples, the stronger presence of *Wolbachia* resulted in a reduction of *Elizabethkingia* (1.8%), *Microbacterium* (0.7%), and *Asaia* (3%) reads, whereas *Serratia* remained stable at 2.6%. In contrast, the two UTH populations showed an increase in *Sphingobium* (3.1%) and *Brevundimonas* (2.5%) sequences compared to the BPI. A more detailed analysis of the laboratory populations revealed that the four BPI populations shared similar bacterial communities (*p* > 0.05), whereas the two UTH populations were significantly different (PERMANOVA, *p* ≤ 0.05; betadisper, *p* ≤ 0.05) (Figure S5).

### 3.3. The Bacterial Community of the UTH Populations Based on the Developmental Stage and the Sex of Adult Flies

Differences between the two UTH populations were recorded for multiple bacterial genera (Figure S6). The first population showed increased relative abundance of eight genera compared to P2, including *Brevundimonas* (4 against 1.1%) and *Serratia* (4.9 against 0.4%). In contrast, UTH_P2 showed an increased relative abundance in only four genera. This was mainly due to a higher *Wolbachia* load than UTH_P1 (80.5 against 70%). *Microbacterium* sequences were identified only in population 1, and *Chryseobacterium* only in population 2 with identical percentages (1.3%).

**Figure 3:**
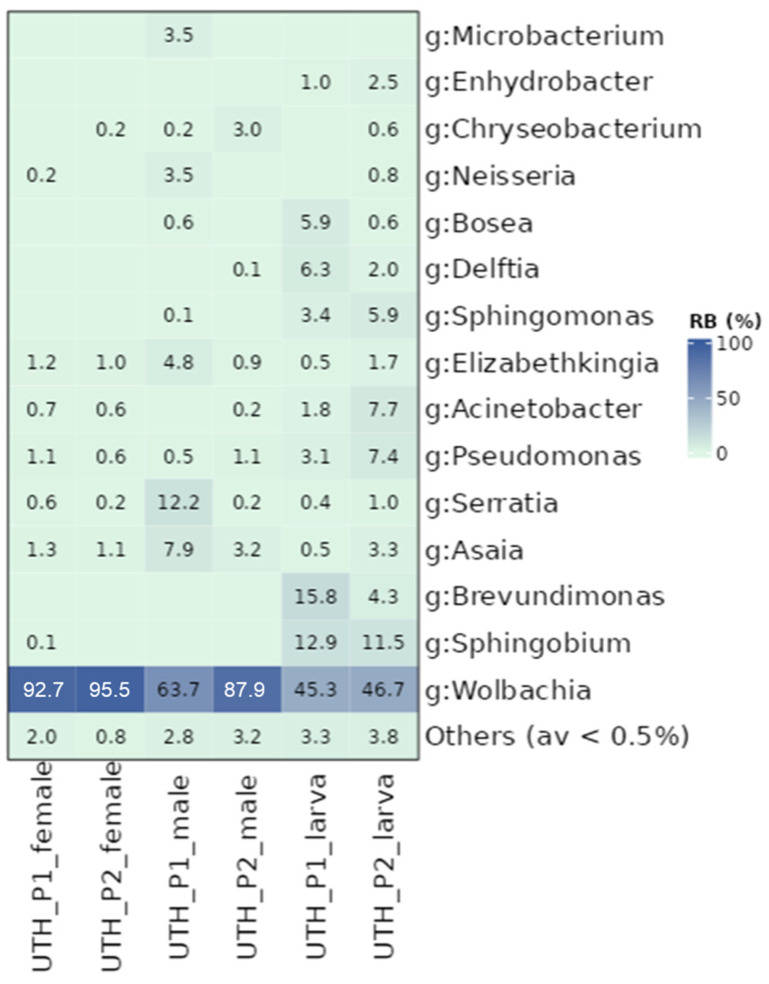
Relative abundance of bacterial genera (g) identified in UTH laboratory populations. Samples are distinguished based on their developmental stage (larvae and adults) and sex (females and males). Other bacterial genera with relative abundance of less than 0.5% were grouped.

Further analysis of the UTH lab populations revealed significant differences between developmental stages as well as between female and male adult flies (Figure S7). Adult mosquitoes were characterized by different bacterial communities compared to their respective larvae (*p* ≤ 0.05). Similarly, the females of each population contained different bacterial communities than the corresponding males (*p* ≤ 0.05). In P1, significant dispersion was observed between males and females (PERMANOVA, *p* ≤ 0.05; betadisper, *p* ≤ 0.05). Generally, females had the highest percentage of *Wolbachia* (average 94.1%) compared to males (75.8%) and larvae (46%) (Figure 3). On average, larvae contained a stable community of *Sphingobium* (12.2%), *Brevundimonas* (10.1%), *Pseudomonas* (5.2%), *Acinetobacter* (4.7%) *Sphingomonas* (4.7%), *Delftia* (4.1%), and *Bosea* (3.3%), which were generally absent from the adult stages. This resulted in larvae containing a richer and more diverse community than the adults (Table S5). Other bacterial genera showed irregular presence between the groups. For instance, *Serratia* reads were found in P1 males with high values (12.2%) compared to all other samples. Although differences were observed between different sexes and developmental stages, it is interesting to note that P1 and P2 larvae shared similar bacterial profiles, and so did P1 and P2 female mosquitoes (*p* > 0.05; Figure S7B).

### 3.4. The Bacterial Community of the BPI Populations Based on the Developmental Stage and the Sex of Adult Flies

**Figure 4:**
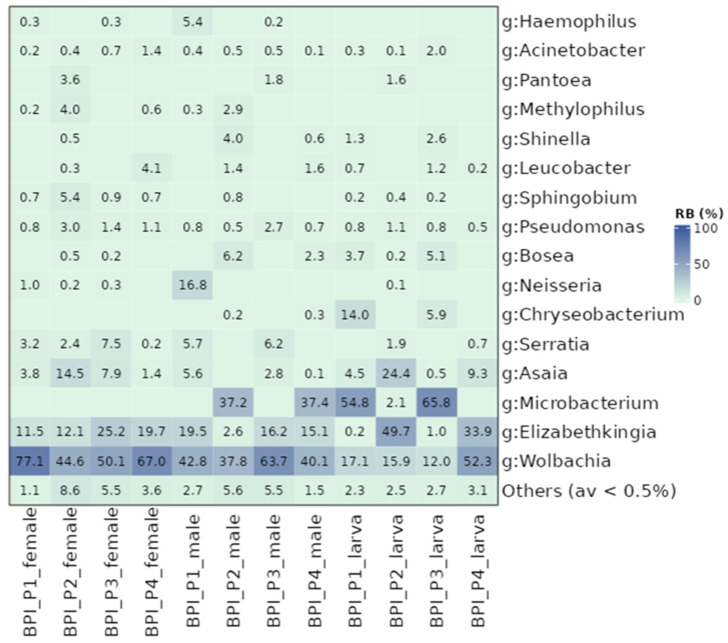
Heat map displaying relative abundance values for BPI laboratory populations. Samples were analyzed based on their developmental stages (larvae and adults) and the sex (females and males). Other bacterial genera with less than 0.5% relative abundance values were grouped.

Although the BPI populations were similar in the structure of their bacterial communities, differences were observed between various subgroups, including the developmental stage and the sex of adult mosquitoes. Adults developed different bacterial communities in most populations compared to larvae (*p* ≤ 0.05; Figure S8A). The only exception was BPI_P4, where larvae displayed similar communities to females but differed from males (Figure S8B). Sex-related differences were observed in P1, P2, and P4, with female and male mosquitoes developing distinct bacterial profiles (*p* ≤ 0.05; Figure S8B). In contrast, only P3 showed similar profiles between males and females (*p* > 0.05; Figure S8B). Regarding alpha diversity metrics, females exhibited higher indices than larvae and males (Table S6). In the case of *Wolbachia*, the general trend observed in the UTH populations was also observed in the BPI populations (Figure 4). Female samples contained the highest titer (59.7% on average), followed by male mosquitoes (46.1%) and larvae (24.3%). Regarding other bacterial genera, *Elizabethkingia* (0.2–49.7%), *Asaia* (0.1–24.4%), *Pseudomonas* (0.5–3%), and *Acinetobacter* (0.1–2%) were present in almost all samples. Unlike the UTH populations, *Sphingobium* was present in both adults and larvae, whereas *Brevundimonas* was absent. *Serratia* was mostly present in adult individuals (3.1%) rather than in larvae (0.7%). Certain bacterial genera, such as *Microbacterium* and *Chryseobacterium*, were absent from female samples, but were identified in larvae and male mosquitoes. *Microbacterium* was the third most abundant genus across all samples, with relative abundance values ranging from 2.1 to 65.8%. Moreover, *Chryseobacterium* was mostly present in larvae (4.8%) whereas males contained, on average, only 0.1%. The genera *Methylophilus* and *Haemophilus* were only present in adult mosquitoes but with low densities, 1% and 0.8%, respectively.

### 3.5. The Bacterial Community of the BPI Blood-Fed Populations

**Figure 5:**
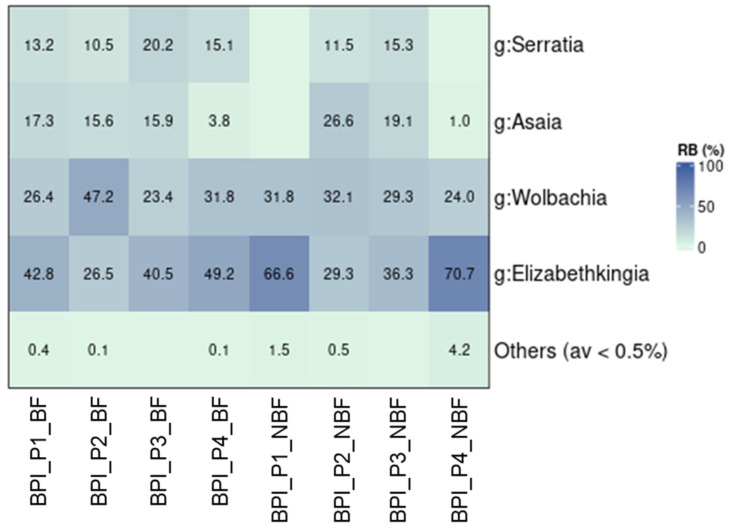
The relative abundance of bacteria found in female mosquitoes from BPI that were offered blood meals (BF) or not (NBF).

Blood feeding influenced the bacterial communities of female mosquitoes to some extent. As mentioned earlier, blood-fed populations were collected only from the BPI (Table 1). These were compared to female mosquitoes that were not provided with blood meals. Significant differences in the composition of bacterial communities were observed between the blood-fed and non-blood-fed groups in two populations, P1 and P4 (*p* ≤ 0.05; Figure S9). In contrast, blood feeding did not affect the bacterial communities of female mosquitoes in P2 and P3 (*p* > 0.05). Generally, alpha diversity indices were similar between the two groups, with only a marginal difference in the Simpson diversity index (Table S7). Contrary to previous findings in the BPI and the UTH populations, *Wolbachia* was not the dominant bacterium in this dataset (Figure 5). It was prevalent only in P2. The dominant genus in the remaining populations was *Elizabethkingia*, with 26.5–70.7% relative abundance. The non-blood-fed groups of P1 and P4 were distinguished from their corresponding blood-fed groups mainly by the absence of *Asaia* and *Serratia* (Figure 5).

### 3.6. The Bacterial Community of Wild Populations

**Figure 6:**
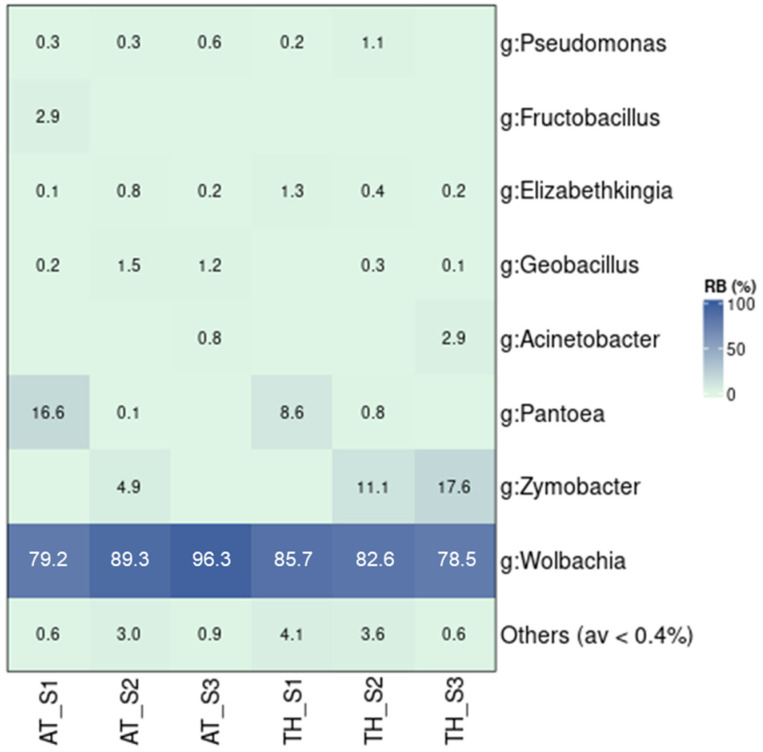
The relative abundance of bacterial communities in wild *Ae. albopictus* samples. The samples were collected in Attica (AT) and Thessaly (TH) in three different time periods. S1: June 2022, S2: August 2022, S3: October 2022.

Wild populations collected in different time periods—June, August, and October 2022 in Attica (AT) and Thessaly (TH)—generally exhibited similar bacterial profiles, characterized by the strong presence of *Wolbachia* (Figure 6 and Figure S10). Only the population collected in Athens in October (AT_S3) showed significant differences compared to the populations collected in Thessaly and the population collected in Athens in June (AT_S1) (*p* ≤ 0.05; Figure S10). On the other hand, AT_S3 was found to host bacterial communities similar to AT_S2. These results can be mostly associated with the high levels of *Wolbachia* found in AT_S3 (96.3%), compared to the populations from Thessaly (82.3%) and AT_S1 (79.2%). Apart from *Wolbachia*, some populations also contained high densities of *Zymobacter* (4.9–17.6%) and *Pantoea* (0.1–16.6%). Moreover, a stable presence of *Geobacillus*, *Elizabethkingia*, and *Pseudomonas* was observed across all samples.

### 3.7. The Presence of Wolbachia in the Laboratory and Wild Populations

**Figure 7:**
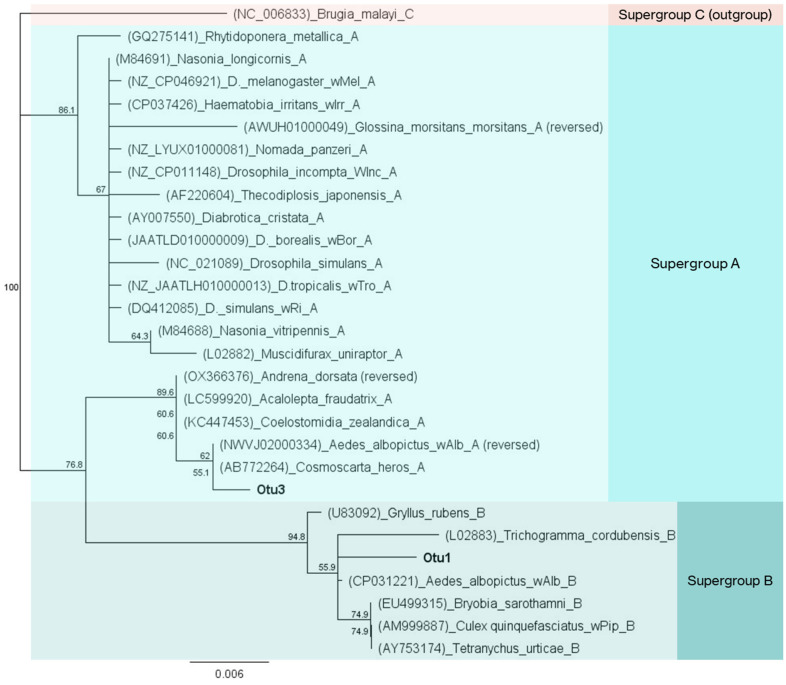
Neighbor-joining tree of *Wolbachia* sequences identified in *Ae*. *albopictus* samples during the high-throughput analysis of bacterial communities. A 350 bp fragment of the 16S rRNA was used for phylogenetic analysis. OTU3 is clustered with sequences in supergroup A, and OTU1 in supergroup B. A supergroup C *Wolbachia* sequence was used as outgroup.

Analysis of the bacterial profiles revealed the presence of two *Wolbachia* operational taxonomic units (OTUs) in *Ae. albopictus* samples, namely OTU1 and OTU3 (Table S3). OTU1 was identified with overall higher relative abundance values than OTU3 (42.2 against 21.5%). Only in two out of twenty populations was OTU3 more abundant than OTU1 (Figure S11). Notably, they were both control populations from the blood meal group (P1_NBF and P2_NBF). Phylogenetic analysis placed OTU3 in supergroup A of *Wolbachia*, close to the reference sequence from strain *w*AlbA of *Ae. albopictus*, whereas OTU1 clustered with strain *w*AlbB from supergroup B (Figure 7). In Attica, the abundance of *Wolbachia* gradually declined from wild to laboratory populations (*p* ≤ 0.05; Figure S11). Conversely, wild and laboratory populations from Thessaly retained the same amount of *Wolbachia* despite the domestication process (*p* = 0.184). *Wolbachia* sequences were detected in all 363 samples used in the survey (100% incidence). A more detailed analysis revealed the presence of both *Wolbachia* OTUs in 362 samples (Table S8). The only exception was a female individual from BPI_P4, where OTU3 (*w*AlbA) was not detected. Various samples also contained a rather low titer (1–10 reads, 0.02–0.2%) of *w*AlbA. In contrast, the lowest recorded concentration of *w*AlbB was 35 reads (0.74%) in a wild individual from the TH_S1 population (collected in June).

### 3.8. Sex-Related Differences in the Presence of the Two Wolbachia Strains

**Figure 8:**
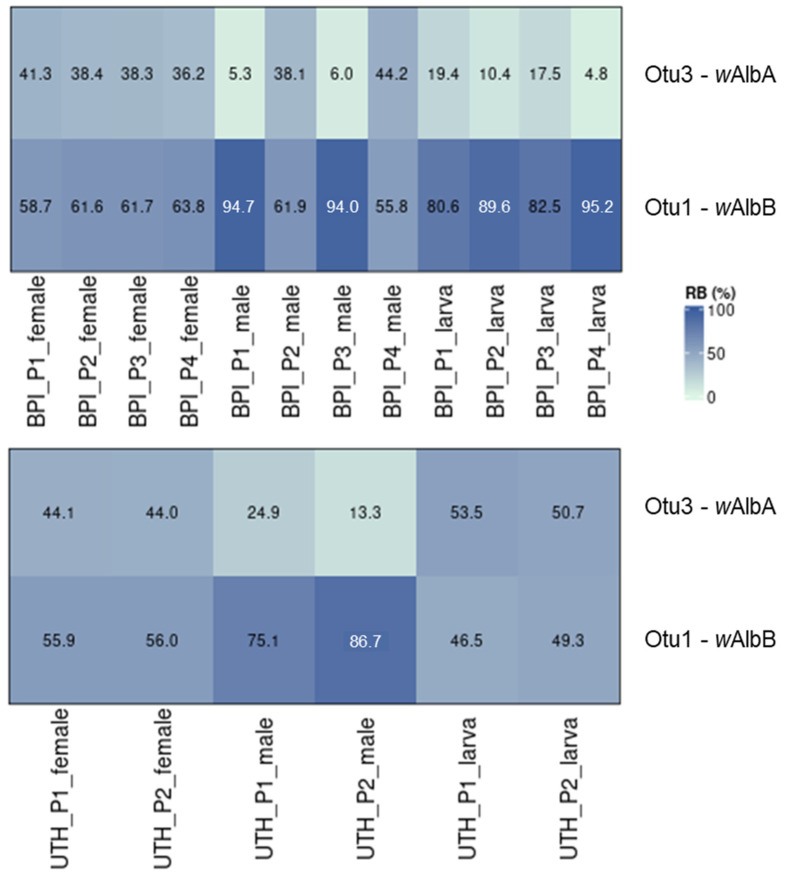
The differential presence of *w*AlbA and *w*AlbB in male and female mosquitoes of the two laboratory populations (BPI and UTH).

A detailed analysis of the two laboratory populations (BPI and UTH) revealed the differential presence of *w*AlbA and *w*AlbB strains in female and male individuals. In general, *w*AlbA sequences were identified at a lower relative abundance in male mosquitoes compared to the females of the same population (Figure 8). This was the case in P1 and P3 from the BPI, as well as in both UTH populations. In the remaining two BPI populations, P2 and P4, both *Wolbachia* strains were identified with similar relative abundances in male and female individuals.

### 3.9. The Core Microbiome of Aedes albopictus Mosquitoes from Greece

**Figure 9:**
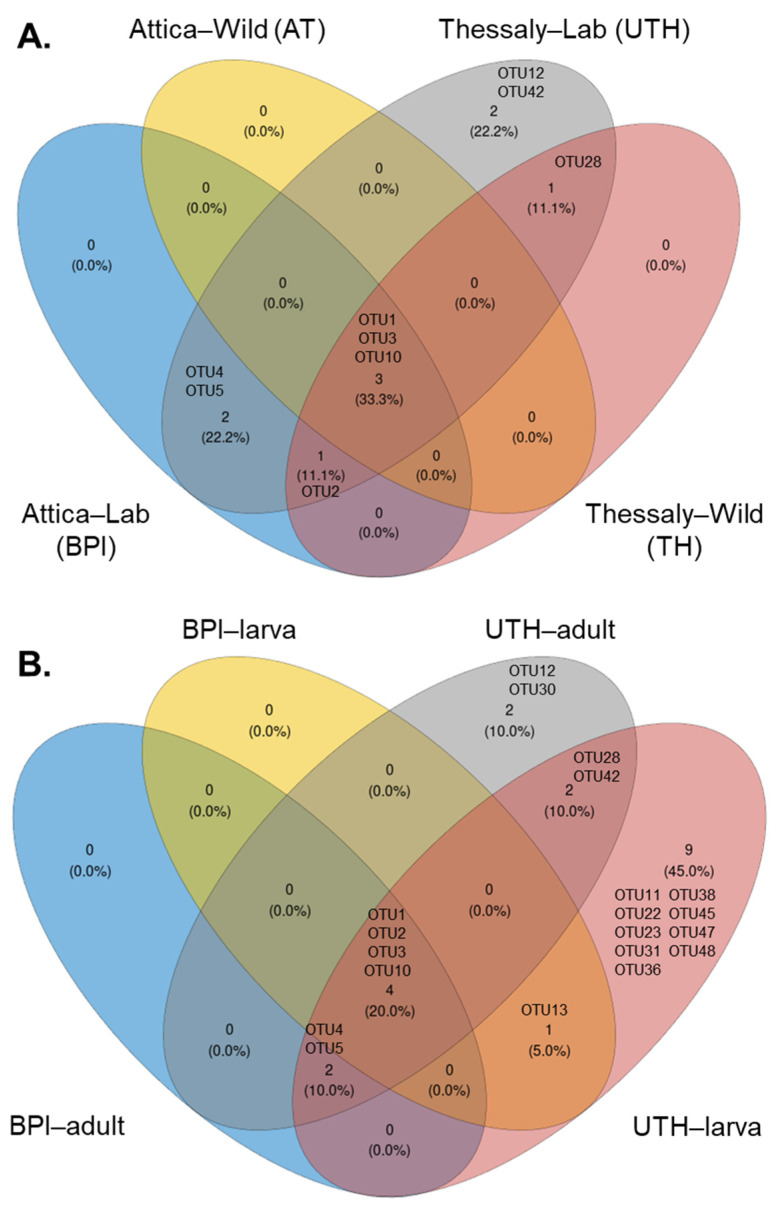
The core bacteria of *Ae*. *albopictus* samples between (**A**) wild and laboratory populations and (**B**) larval and adult laboratory samples.

The core microbiome was examined between the wild and laboratory populations and between adults and larvae of the laboratory populations. As expected, the two *Wolbachia* OTUs, OTU1 and OTU3, were ubiquitous in almost all samples and were considered part of the insect core microbiome. Only one additional OTU (OTU10), classified as a *Pseudomonas* species, was found in all the populations (Figure 9 and Figure S10, and Table S10). *Elizabethkingia ursingii* (OTU2) sequences were identified in all populations from Thessaly and in laboratory populations of Attica (Figure 9A). This OTU was also present in wild populations from Attica, but due to its low incidence (71% of samples), it was not identified as a core member. Nevertheless, it was detected in the adult and larval stages of the laboratory populations (Figure 9B).

Two OTUs were predominantly found in the laboratory populations, *Serratia marcescens* (OTU4) and *Asaia siamensis* (OTU5). More specifically, in the UTH group they were recognized as core members in both adults and larvae, whereas in the BPI group they were only recognized in adult samples (Figure 9B). A species belonging to the genus *Cutibacterium* (OTU28) was shared between laboratory and field-caught samples from Thessaly. In the lab samples, it was present in both adults and larvae. *Phyllobacterium* sp. (OTU12) and *Vibrio metschnikovii* (OTU42) were recognized as members of the core bacteria that compose the communities of laboratory samples from Thessaly. In this case, the *Phyllobacterium* strain was a core member of adult mosquitoes, and *Vibrio metschnikovii* of larvae. Adult mosquitoes from UTH contained an additional core bacterium, *Aeromonas* sp. (OTU30). Interestingly, larvae from both lab facilities shared a common bacterium identified as *Acinetobacter johnsonii* (OTU13). However, no common OTUs were observed between adult samples from the two laboratories. Finally, larvae from UTH contained a unique consortium of bacteria, including *Bosea* (OTU11), two *Sphingomonas* species (OTU22 and OTU47), *Sphingobium* (OTU23), *Brevundimonas* (OTU31), *Rhizobium* (OTU36), *Delftia* (OTU38), *Pseudomonas* (OTU45), and *Enhydrobacter* (OTU48).

### 3.10. The Culturable Bacterial Diversity of Laboratory Larvae and Adult Mosquitoes

**Figure 10:**
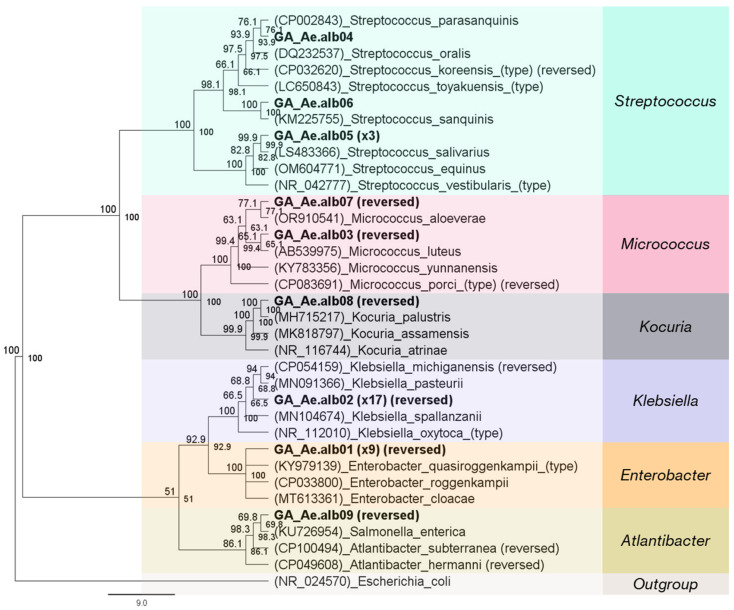
Neighbor-joining tree of 16S rRNA sequences of bacteria isolated from adult mosquitoes. Almost the full 16S rRNA gene (~1300 bp) was used for the development of the tree. Isolates are highlighted in bold letters. Parentheses at the start of each reference sequence denote their accession number.

In total, 17 and 35 morphologically distinct colonies were picked from plates inoculated with larval and adult extracts, respectively. Selected colonies were isolated from all three media (LB, PDA, and MacConkey agar) (Table S11). In the case of the larval extract, most of the strains were isolated from MacConkey and PDA agar plates, while for adult mosquitoes they were isolated from LB agar. Fourteen larval isolates were identified as *Aeromonas hydrophila* (82.4%), and three were placed in the *Acinetobacter* cluster (17.6%) with the 16S rRNA sequences of *A. beijerinckii* and *A*. *calcoaceticus*, which are identical (Figure S12). More diverse bacterial isolates were detected in adult mosquitoes. From these, seventeen were clustered with *Klebsiella* 16S rRNA sequences (48.6%) and nine with *Enterobacter* (25.7%) (Figure 10). The other strains were classified as *Streptococcus* (14.3%), *Micrococcus* (5.7%), *Kocuria* (2.9%), and *Atlantibacter* (2.9%). The derived sequences from all isolated strains showed more than 98% (98.2–100%) sequence similarity with their reference sequences.

## 4. Discussion

### 4.1. The Bacterial Communities of Aedes albopictus Based on the Studied Population Parameters

Laboratory-reared and wild *Ae. albopictus* from Greece exhibited differentially structured bacterial communities. This was also the case for laboratory and field-caught *Ae. albopictus* originating from Brazil [71], and Italy [72], although cases of similar communities between wild and lab mosquito populations have also been reported [73]. In terms of alpha diversity, laboratory samples contained richer and more diverse profiles than wild samples. Opposite trends were generally observed in previous studies. Field-collected larvae from Italy were more diverse than lab-reared larvae, but adult lab mosquitoes showed slightly higher or similar diversity metrics to their wild counterparts [73]. In Brazil, newly domesticated lab samples (F_1_ generation) showed lower diversity compared to both wild mosquitoes and an older lab strain (>F_30_ generation). The controlled rearing conditions in laboratory environments, including artificial diets and housing, have a profound impact on insect microbiota, leading to either an increase or decrease in community diversity [74,75,76,77]. Variations have even been recorded within the same facility over time. For example, newly domesticated *Ceratitis capitata* larvae (F_0_ generation) maintained the same gut diversity in the first laboratory generation (F_1_). Although a significant decrease was observed during the second generation (F_2_), the bacterial community quickly returned to its initial levels in subsequent generations (F_4_–F_13_) [74]. The discrepancy observed in alpha diversity metrics between different studies could also be related to differences in the experimental setups. These may include filtering reads of dominant genera (e.g., *Wolbachia*) and focusing on specific developmental stages, sexes, tissues or other factors [71,73].

The composition of the bacterial communities of both laboratory-reared and wild-caught *Ae. albopictus* mosquitoes from Greece was characterized by a strong presence of Proteobacteria (81.4%), especially Alphaproteobacteria (71.5%). The dominance of Proteobacteria was also reported in the microbiota of both laboratory and wild *Ae. albopictus* populations collected worldwide, primarily due to the high prevalence of *Wolbachia* [15,41,71,72,73,78,79,80,81,82,83,84,85,86,87,88,89,90]. Exceptions to this rule were observed when focusing on the gut tissue, at specific developmental stages (e.g., larvae) or on blood-fed females. In these cases, other bacterial phyla were prevalent, like Bacteroidota (e.g., *Elizabethkingia*) [15,91]. The other phyla identified in the studied samples included Bacteroidota (Bacteroidetes) (12%), Actinobacteriota (5.9%), and Firmicutes (0.67%). These bacteria were also common components of bacterial assemblages of *Ae. albopictus*, found in varying concentrations. For instance, Firmicutes have been identified with low (0.51–3.7%) [41,73,78,89] or relatively high (10.3–27.2%) [81,82] relative abundance. Together with Proteobacteria, these four phyla constituted almost 99% of the total bacterial community in adult *Aedes* mosquitoes [18].

*Wolbachia* was the main component of the bacterial communities in both laboratory and wild *Ae. albopictus* mosquitoes. It was identified in all 363 samples (100% incidence) with an average relative abundance of 63.7%. Similarly, high *Wolbachia* infection rates (87–100%) were identified in previous studies of Greek populations of *Ae. albopictus* [39,40]. The dominance of *Wolbachia* was previously observed in amplicon metagenomic surveys, which employed different experimental setups. These studies focused on the gut tissue of wild females [85], on the reproductive tissue of adult *Ae*. *albopictus* mosquitoes [17], or on whole adult individuals from laboratory colonies and wild populations [41,82,83,84,87]. However, *Wolbachia* infection rate in *Ae. albopictus* varied greatly among populations worldwide, ranging from 11% to 100% [25,92,93]. In this survey, *Wolbachia* was identified with two OTUs corresponding to the *w*AlbA and *w*AlbB strains, which are commonly found in *Ae. albopictus* mosquitoes [87,89,94,95]. These strains were also confirmed by PCR screening and MLST genotyping in Greek *Ae*. *albopictus* populations [40]. *Wolbachia* interacts negatively or positively with other members of the insect microbiome, significantly influencing its structure and composition. The mechanism of these interactions can be direct or indirect. Direct interactions may involve the release of effectors or competition for available niches or resources, while indirect interactions may occur through the modulation of the host immune system [96,97,98,99]. Typical examples include *Wolbachia’s* interactions with *Asaia* and *Spiroplasma* [100,101]. The bacterial communities were further characterized by the presence of other bacterial genera, including *Elizabethkingia*, *Serratia*, *Asaia*, *Microbacterium*, *Pseudomonas*, *Chryseobacterium*, *Sphingobium*, *Brevundimonas*, and *Acinetobacter*, all of which are frequent members of the microbial communities of *Aedes* mosquitoes [16,18]. The specific functional roles of these symbiotic bacteria are often influenced by tissue localization and have not been fully explained. They may contribute to nutrition, maintain the stability of the microbial community, interact with other members, interfere with pathogen transmission, and neutralize toxic substances [18].

Variability was also observed in the structure of the bacterial communities between the two laboratory facilities at the BPI and the UTH. This variability was mainly due to *Wolbachia*’s low presence in the BPI samples. This reduction in relative abundance could be related to the housing conditions of larvae, as *Wolbachia* may become scarcer owing to crowding [102]. Although both facilities used similar rearing protocols, the larval densities differed. The UTH used moderately crowded conditions, with 750 larvae per liter of rearing water, whereas larvae from the BPI were raised at a density of 2000 larvae per liter. Moreover, the difference in bacterial communities could be explained by the age of the populations, as those from the BPI were newly domesticated compared to the populations from the UTH, which were maintained for more than five generations under artificial rearing conditions. Variations in other taxa included an increase in the relative abundance of *Microbacterium* and *Elizabethkingia* within the BPI populations that proliferated in the absence of *Wolbachia*. At the same time, the structure of the bacteriome was similar among the four populations reared in the BPI and differed between the two populations reared in the UTH. However, the differences in UTH were not extended and could be attributed only to male individuals (UTH_P1_male and UTH_P2_male), as females and larvae shared similar community structures. Therefore, laboratory mosquito populations within the same facility, either newly domesticated or older, developed similar bacterial profiles even though the samples were collected from geographically remote areas in the country. The same was observed by Minard et al., who collected mosquitoes from even more remote areas, such as La Réunion and Montpellier [103].

Extensive differences in community structure were observed within each population based on the developmental stage and sex of the flies. This is generally an expected outcome, as males and females are usually characterized by the differential presence of *Wolbachia*. Females generally display higher infection rates and density than males [30], as the bacterium is mostly associated with ovaries rather than testes in *Ae. albopictus* [17]. However, Lin et al. [82] observed a similar bacterial community structure between whole male and female *Ae. albopictus*, despite differences in the relative abundance of *Wolbachia* (37.7 in males and 78.7% in females). The reduction in the relative abundance of *Wolbachia* recorded in five male groups was followed by an increase in various gut bacteria, including *Serratia*, *Asaia*, *Elizabethkingia*, *Neisseria*, *Microbacterium*, and *Chryseobacterium*. Differences observed across developmental stages were caused not only by the differential presence of *Wolbachia* but also by additional bacterial genera. In this regard, larvae from both facilities contained less *Wolbachia* than adults, a trend seen previously in *Ae. albopictus* [73,86,89]. Larvae from the UTH were distinguished from the adults by their richer and more diverse communities, which were composed of *Sphingobium*, *Brevundimonas*, *Sphingomonas*, *Pseudomonas*, *Acinetobacter*, *Delftia*, and *Bosea*. On the other hand, larvae from the BPI contained higher titer of *Microbacterium*, *Asaia*, *Chryseobacterium*, and *Bosea*, whereas adults were characterized by a stronger presence of *Serratia*, *Methylophilus*, and *Haemophilus*. The differences observed in the bacterial profiles between adult mosquitoes and larvae can be mostly attributed to the unique habitats and feeding requirements of each life stage, with the aquatic environment shaping the larval microbiota and the terrestrial environment the bacterial communities of adults [14,16,18,41,79].

The blood-fed females from BPI showed mixed trends in the structure of their bacterial profiles, with two populations containing similar profiles and two populations having different profiles compared to their non-blood-fed counterparts. Blood meals have been shown to affect the mosquito microbiome to varying degrees, resulting in slight or significant modifications [15,104,105]. Notably, in our samples, the prevalent bacterium in all three populations, except for P2, was *Elizabethkingia* and not *Wolbachia*. This was true for both non-blood and blood-fed samples. *Serratia* and *Asaia* were the only remaining genera present in most of the samples with relatively high densities. These bacteria, which are mostly associated with the gut [16], were not detected in the non-blood-fed populations P1 and P4. This was the main reason that the bacterial profile of these two populations differed from their corresponding blood-fed samples. *Elizabethkingia* has been previously identified in both blood and non-blood-fed samples [15,104,105]. For example, high densities were found in gut samples of teneral females, as well as in sugar-fed and blood-fed females from an F_13_ lab population [15]. In this case, the bacterium showed the highest relative abundance in blood-fed females [15]. Similarly, it was found to be highly prevalent in gut samples extracted from blood-fed females of an F_15_ lab population [104], as well as in larvae from a laboratory colony that was maintained for several generations [72]. *Elizabethkingia* was identified in both blood and non-blood-fed individuals in all previous cases. Moreover, it was generally prevalent in blood-fed females and well-established laboratory colonies. Our data also supports the presence of *Elizabethkingia* in both types of samples. However, we recorded different trends in the relative abundances of non-blood-fed samples, which contained higher densities of the microbe. In truth, this discrepancy is mostly associated with the absence of *Asaia* and *Serratia* from samples P1 and P4, as *Wolbachia* densities remained stable in all samples regardless of the diet. Notably, a reduction in the relative abundance of *Elizabethkingia* as a result of blood meal provision was previously recorded in *Anopheles gambiae*, which were bred in semi-natural habitats [18,106]. This could also have been the case with samples P1_BF and P4_BF, as they displayed a reduction in *Elizabethkingia* and an increase in *Serratia* and *Asaia* compared to their non-blood-fed controls. This reduction due to blood feeding may have left space for the proliferation of *Serratia* and *Asaia* in the guts of these samples. In contrast, *Elizabethkingia* was not affected by blood feeding in P2_BF and P3_BF. As a bacterium that is mostly associated with gut tissue, *Elizabethkingia* is thought to be implicated in the provision of nutrients to the mosquito by participating in erythrocyte lysis [79,107,108]. Blood feeding favored an increase in the relative abundance of *Serratia*, and to a lesser degree *Asaia*. The relative quantity of *Asaia* has been shown to increase significantly 72 h after a blood meal in *An. stephensi* [109]. In our study, although *Asaia* was present in all four blood-fed populations and absent in two non-blood-fed populations, its average relative abundance was similar between the two groups. In the case of *Serratia*, it has been suggested that it possesses hemolytic activities and is involved in blood digestion in *Ae. aegypti* [110]. Therefore, the increase in blood-fed females seems justified., A similar nutritional role has been suggested for *Asaia* based on observations in *An*. *stephensi* larvae [111]. *Serratia* and *Asaia* have been previously identified in the gut tissues of non-blood-fed and blood-fed female *Ae. albopictus* [112]. *Asaia* showed a higher prevalence in the non-blood-fed group and *Serratia* a slightly higher prevalence in the blood-fed group. Interestingly, apart from their nutritional role, *Serratia* strains differentially affect the vectorial capacity of mosquito species, decreasing it in the case of *Plasmodium* [113] and increasing it in the case of the dengue virus [114].

The structure of the communities of the wild populations followed a similar pattern to that of the laboratory populations, with most bacterial profiles containing similarities rather than differences. These samples were collected from two sites in Attica and Thessaly at different times in June, August, and October 2022. Therefore, they represent the temporal evolution of the bacterial communities of the studied populations when mosquitoes are active in Greece. The results showed that the bacterial communities remained relatively stable over a five-month period within a certain area. This outcome could be attributed to the high presence of *Wolbachia* in all the samples, combined with the stable but low presence of certain genera, such as *Geobacillus*, *Elizabethkingia*, and *Pseudomonas*, that are commonly found among the microbiota of various mosquito species [14,18]. In the case of *Elizabethkingia*, the bacterium was previously found to negatively correlate with *Wolbachia* in whole-body samples of females, and its relative abundance in these samples was low [82]. Despite the general uniformity in wild populations, interesting small-scale variations were still observed. These included the presence of *Pantoea* in both populations in June (AT_S1 and TH_S1), and its substitution by *Zymobacter* in August and October in both populations of Thessaly, but only in AT_S2 (August). The lack of *Zymobacter* and disproportionally higher presence of *Wolbachia* in the sample collected from Attica in October (AT_S3) were the main reasons why this population differed significantly from the rest (except for AT_S2). The presence of *Pantoea* at the start of the active period of *Ae. albopictus* in Greece could highlight an important nutritional role of this microbe. Indeed, *Pantoea* has been linked to increased insect fitness through the metabolism of compounds, and even toxic substances [81,115]. As the summer period progressed, *Pantoea* was replaced by *Zymobacter*, which is probably more abundant in the available diet and undertakes a similar nutritional role. *Zymobacter*, a plant bacterium associated with palm sap, is also a transient member of the community acquired through feeding [41]. Although little is known about the exact role of *Zymobacter*, it has been previously identified in *Ae*. *albopictus* [41,80,81]. AT_S3 mosquitoes lack both *Pantoea* and *Zymobacter*, therefore their role could be fulfilled by other bacterial genera present, such as *Acinetobacter*, *Geobacillus*, *Elizabethkingia*, and *Pseudomonas*. Even by excluding *Wolbachia* reads from the analysis, an effect of geography on the remaining bacterial components could not be observed between wild populations from Thessaly and Attica. Nevertheless, variation in the microbiota due to geography or different habitats is usually common in insects and has been previously recorded in *Ae. albopictus* [116,117].

As expected, *Wolbachia* and *Elizabethkingia* OTUs were among the core bacteria of *Ae*. *albopictus* populations from Greece, showing high incidence and prevalence. The same was true for *Pseudomonas*, although it was mostly identified with low relative abundance. Along with *Serratia* and *Asaia*, which were found in all the laboratory populations regardless of geographical origin, these genera constituted the core microbiota of the studied populations. Additional bacteria were also present in the groups. For example, *Cutibacterium* was found in both wild and laboratory samples from Thessaly and, more specifically, in lab-reared adults and larvae. This bacterium was identified to have a high relative abundance in the gut tissue of permethrin-resistant female *Ae. aegypti* from an F_22_ lab strain [118], but also in natural habitats of *Aedes* mosquito larvae [119], suggesting that it is stable in both natural and laboratory setups, in both adults and larvae. *Phyllobacterium* and *Aeromonas* strains were present in UTH adult samples. *Aeromonas* is a common finding in adult mosquitoes and larvae, often identified with a high relative abundance, higher than *Wolbachia* or *Elizabethkingia* [15]. It can associate with the gut and support mosquito development [14,120], or enhance oviposition [16]. Notably, an *Acinetobacter* OTU was part of the core bacteria of larvae from both laboratories, suggesting a possible role for the bacterium during larval development. In this regard, and similarly to *Aeromonas*, an *Acinetobacter* strain rescued the development of axenic *Ae. aegypti* larvae [120]. Moreover, *Acinetobacter* strains are found not only in adult mosquitoes and larvae but also in their natural habitats [14,15,121]. Although the core microbiota seems to differ between mosquito populations worldwide, some of these genera, such as *Acinetobacter*, *Aeromonas*, *Wolbachia*, and *Pseudomonas*, have been previously identified as prominent members [14,16,71].

The identified cultivable diversity consisted of bacteria that are among the typical components of *Ae. albopictus* symbiotic communities [14,15,18,117,122,123,124]. Most isolated strains belonged to the Proteobacteria, including *Aeromonas*, *Acinetobacter*, and members of the Enterobacteriaceae, such as *Atlantibacter*, *Enterobacter*, and *Klebsiella*. The cultivable diversity also included members of Actinobacteria and the family Micrococcaceae, like *Kocuria* and *Micrococcus*, as well as Firmicutes (*Streptococcus*). Various *Aeromonas*, *Acinetobacter*, and *Enterobacter* strains have been previously isolated from homogenates of both wild and laboratory adult mosquito samples [15]. *Acinetobacter* is a common cultivable genus found both in larvae and adult *Ae. albopictus* [15,95,122,124], although in our case it was isolated only from larval homogenates. *Klebsiella* and *Kocuria* isolates have been found in both larvae and adults [122,124], while *Micrococcus* is only in the midgut of adult mosquitoes of both sexes [124]. Isolated strains such as *Enterobacter*, *Klebsiella*, *Aeromonas*, and *Acinetobacter* are further being analyzed through whole-genome sequencing to characterize their functional properties. As strains from similar genera have already been found to enhance various fitness parameters when used as dietary supplements in laboratory-reared insects for SIT applications [7,11,19,125,126], these isolates could also be exploited for this purpose if deemed suitable.

Amplicon sequencing analyses often exhibit biases in the representation of microbial communities due to multiple factors. These include different DNA extraction efficiencies among bacterial taxa, primer sensitivity and specificity, the representation of reference sequences in databases, and variations in the number of 16S rRNA gene copies per bacterial taxon [127,128,129,130]. In the present study, thorough surface sterilization of samples and the use of negative PCR controls (no band detection while visualizing the whole volume of the reaction) were used to minimize the effect of contamination. Notably, a comparative study of surface sterilization methods found no significant changes in symbiotic bacterial communities even in the absence of surface sterilization [131]. Furthermore, during data analysis, singletons and bacterial taxa with relative abundance lower than 0.1% were removed. The strict filtering process may have resulted in the loss of rare or underrepresented taxa but ensured that contamination was minimized. As with all culture-dependent and high-throughput analyses, and despite the use of negative controls, proper handling, and thorough sterilization procedures, it can never be excluded that some of the observed diversity is due to contamination since certain bacterial genera, such as *Kocuria*, *Micrococcus*, *Microbacterium*, *Phyllobacterium*, and *Streptococcus* are included in the list of common laboratory contaminants [132].

### 4.2. The Prevalence of Wolbachia in the Bacterial Communities of Aedes albopictus Mosquitoes

Despite the strong presence of *Wolbachia* in the dataset, its concentration displayed a certain degree of heterogeneity, which can be attributed to various factors, including the developmental stage or sex of the mosquitoes. In this study, adult individuals contained more *Wolbachia* sequences than larvae in five of the six studied laboratory populations, with the exception of larvae and males of BPI_P4. As mentioned earlier, this trend was frequently observed in studies and could be attributed to the distinct ecosystems in which the larval and adult stages live [14,73,86,89]. In this regard, previous analyses of water samples from *Ae. albopictus* larval ecosystems either detected *Wolbachia* with very low densities or not at all [73,79,89]. Even though the presence of *Wolbachia* was not as strong as in the adult samples, the studied larvae still maintained a considerable percentage of *Wolbachia* (12–52.3%), probably of maternal origin, since the bacterium is generally transmitted vertically [26].

Sex-related differences in the distribution of *Wolbachia* were also observed, with females carrying more *Wolbachia* than males. This was the case for almost all laboratory populations except for BPI_P3 males. Notably, in this population, female samples exhibited higher densities of *Asaia* than male samples (7.9% vs. 2.8%). The strong presence of *Asaia* in these samples may have resulted in increased competition with *Wolbachia* strains for localization in the ovaries, despite *Asaia* being mostly associated with the gut tissue of *Ae*. *albopictus* mosquitoes [17,101,133]. The stronger presence of *Wolbachia* in females than in males has been previously recorded in various *Ae. albopictus* populations [17,30,41]. Interesting information was also obtained regarding the incidence and relative abundance of the two *Wolbachia* strains, *w*AlbA and *w*AlbB, in male and female samples. As mentioned previously, 362 samples carried double infections, except for one female individual of BPI_P4 that carried only *w*AlbB sequences. In agreement with this finding, several studies have reported a high incidence of double infections in both sexes [30,95,102]. The high occurrence of superinfected females is quite typical in *Ae. albopictus* [30,84,91,95,134,135]. However, this is generally not the case for male samples, as a significant proportion of them harbor single *w*AlbB infections when screened with PCR techniques [30,40,73,135,136,137]. This lack of detection is usually a result of the low sensitivity of standard PCR assays, as more sensitive approaches, such as qPCR [30,102,138] and semi-nested PCR [137], manage to identify low titer *w*AlbA infections in superinfected males. Similarly, in our case, the increased sensitivity of high-throughput sequencing was able to identify low-titer *w*AlbA infections in males. Indeed, 17 out of 77 male individuals that carried both strains contained *w*AlBA with less than 1% relative abundance. This translates into fewer than 50 16S rRNA sequences that may not be detectable by standard PCR screening and may result in the identification of only *w*AlbB infections in the sample. Overall, the low prevalence of *w*AlbA in male *Ae. albopictus* mosquitoes is a common finding among studies focusing either on *Wolbachia* or the complete bacterial community [30,40,135,138]. The analysis across the sample groups yielded the same outcomes. The female sample groups contained both strains in relatively similar concentrations (Figure 8), whereas four out of six male sample groups showed increased relative abundance of *w*AlbB (75.1, 86.7, 94, and 94.7%) and reduced values of *w*AlbA. The decline in the presence of *w*AlbA is justified because single-infected males with *w*AlbB or males without any infection can still produce viable offspring when crossed with double-infected females [30,40]. The decline in *w*AlbA in males can also be attributed to adaptive mechanisms, as rare occasions of females uninfected with the strain can be found within a population (as in our study) resulting in incompatible crosses [30]. At the same time, the strong presence of strain *w*AlbB in both sexes can be either related to the fact that the infection is more recent or that its vertical transmission and proliferation are more efficient compared to *w*AlbA [30,40,102]. Interestingly, in two populations from the BPI (BPI_P2 and P4), male mosquitoes contained both *Wolbachia* strains with similar frequencies. These groups were characterized by the high presence of *Microbacterium* sequences (OTU6) but were almost devoid of *Asaia* (OTU5). The persistence of *w*AlbA in these male samples could be associated with a positive interaction with *Microbacterium* or the absence of *Asaia*, which might otherwise exclude *w*AlbA from the testes [101,133]. In the remaining four male populations that contained low densities of *w*AlbA, *Microbacterium* was generally undetected (only UTH_P1 males contained 3.5%), and *Asaia* existed with relatively high densities, which were generally higher than the superinfected females of each population (except for BPI_P3). Moreover, these four male groups were characterized by a relatively strong presence of *Serratia* (except for UTH_P2). In *Ae. albopictus*, *Serratia* co-occurred with *Wolbachia* in the testes, was a stable component of male and female guts, and dominated the microbiota of female salivary glands [16,17]. The increased presence of *Serratia* in male samples with low *w*AlbA titers could be related to its proliferation in the testes, but it is difficult to discern whether this change is an effect of *w*AlbA depletion or one of the causes.

Overall, *Wolbachia* sequences were more abundant in wild populations (85.3%) than in the laboratory (53.1%). This outcome was mainly due to the significant differences observed between wild (AT) and laboratory populations from Attica (BPI), with the latter containing lower titers of the bacterium. In contrast, wild samples from Thessaly (TH) contained *Wolbachia* concentrations that were similar to the laboratory samples from the same region (UTH). Since lab populations from Attica originated from newly introduced eggs in laboratory-rearing conditions, it is possible that the domestication process exerted high pressure on *Wolbachia*, resulting in its decline. As mentioned earlier, this result could also be attributed to crowding, as BPI larvae were grown in more crowded conditions than UTH (two against 0.75 larvae/mL) [102]. A similar trend was observed in samples from Brazil, with field-caught samples containing more *Wolbachia* than laboratory strains [71]. *Wolbachia* densities did not seem to follow a specific pattern between laboratory and wild samples. For instance, Coon et al. [79] and Hegde et al. [80] identified similar frequencies between laboratory- and field-collected *Ae. albopictus* samples. This result is consistent with that recorded in our populations from Thessaly. Concurrently, contrasting results were observed in samples from Italy, where five wild adult samples harbored no or very low *Wolbachia* (average 37%) compared to nine laboratory samples (average 77%) [73]. Overall, it appears that variation in the prevalence of *Wolbachia* is a common characteristic of both wild and laboratory populations. In the wild populations, both *Wolbachia* strains were identified with high relative abundance at both sites. As in previous attempts with IIT approaches in *Ae*. *albopictus*, designing an IIT strategy against local populations would require the use or the development of a triple-infected strain [138,139]. The presence of males with low infections of *w*AlbA or *w*AlbB at both sites may increase the risk of population replacement with the newly infected strain. Therefore, combining IIT with SIT to minimize the chances of releasing fertile females infected with the third strain might be the most effective approach for managing *Ae*. *albopictus* populations in the Greek region. Recent advancements in sex sorting with automated sorters and artificial intelligence have enhanced standalone IIT application by minimizing the accidental release of transinfected females, which could replace naturally occurring strains [36,140].

## 5. Conclusions

The continuous accumulation of data using both deep sequencing and culture-dependent approaches creates expectations regarding the potential use of the microbiota of *Ae. albopictus* in vector disease control directly (e.g., *Wolbachia*-mediated CI) or by supporting approaches such as SIT. In this context, our work focused on the detailed characterization of the bacterial diversity of laboratory-reared and field-caught Greek populations of *Ae. albopictus*. The analysis compared the general structure and detailed composition of the bacterial communities considering a variety of parameters, including the origin and temporal evolution of the populations, the effect of domestication and laboratory rearing, the developmental stage, and the sex of the mosquitoes. Notably, different bacterial profiles were developed between the wild and laboratory samples, as well as between samples from different facilities. Different communities also developed between larvae and adults, as well as between males and females. In contrast, generally similar bacterial profiles were observed among the populations within each laboratory facility and the wild populations within each collection site. *Wolbachia* was the dominant genus on most occasions, followed by other common bacterial genera, such as *Elizabethkingia*, *Asaia*, *Serratia*, *Pseudomonas*, *Sphingobium*, *Brevundimonas*, and *Acinetobacter*. An increase in the average relative abundance of *Serratia* was observed across blood-fed populations. In wild populations, apart from the high presence of *Wolbachia*, a shift in the bacterial community was observed between samples collected at different time points, with *Pantoea* succeeded by *Zymobacter*. Ongoing and future analyses are focusing on the characterization of the composition of the microbiota of *Ae. albopictus*, but also seek to further decode its functional role, which remains largely unexplored. Revealing the functional traits of the microbiota will likely provide valuable solutions in the context of the mass-rearing process of *Ae. albopictus* and the containment of vector-borne diseases.

## Figures and Tables

**Table 1:** The samples used for 16S rRNA amplicon sequencing analysis of the bacterial communities in *Ae*. *albopictus* mosquitoes. A total of six laboratory populations were surveyed. Each sample consisted of a pool of five individual mosquitoes. BPI_P: Benaki Phytopathological Institute population. UTH_P: University of Thessaly population.

	Developmental Stage and Sex									
Population	Third Instar Larvae	Fourth Instar Larvae	1-Day-Old		3-Day-Old		14-Day-Old		14-Day-Old Female	
			Male	Female	Male	Female	Male	Female	Blood-Fed (BF)	Non-Blood-Fed (NBF)
BPI_P1	3	4	4	4	3	4	4	3	5	5
BPI_P2	4	3	4	4	4	4	4	4	4	5
BPI_P3	4	4	4	4	4	4	4	4	5	5
BPI_P4	4	4	4	4	4	4	4	4	5	5
BPI samples	15	15	16	16	15	16	16	15	19	20
UTH_P1	5	5	5	5	5	5	5	5	-	-
UTH_P2	5	5	5	5	5	5	5	5	-	-
UTH samples	10	10	10	10	10	10	10	10	-	-
Total no. of samples	25	26	26	26	25	26	26	25	19	20
	243									

## Data Availability

Raw data from this study were deposited to the NCBI SRA archive under accession number PRJNA1177906. The derived 16S rRNA sequences were deposited into GenBank under accession numbers PQ505081-PQ505091 and PQ505106-PQ505107.

## Supplementary Materials

The following supporting information can be downloaded at: https://www.mdpi.com/article/10.3390/microorganisms13071486/s1, Figure S1: Outline of potential uses of the insect microbiome in mass-rearing and vector-borne disease control; Figure S2: The bacterial composition at the phylum level (p) of all the sample groups used in the 16S rRNA amplicon survey; Figure S3: The bacterial communities at the genus level (g) of laboratory-reared and wild populations of *Ae*. *albopictus* mosquitoes; Figure S4: Relative abundance of bacterial genera in the BPI and UTH laboratory populations; Figure S5: The structure of the bacterial communities of lab populations; Figure S6: Relative abundance of bacterial genera in the laboratory populations from UTH; Figure S7: Community structure of UTH laboratory populations according to the developmental stage and the sex of the flies; Figure S8: Pairwise comparison of mosquito bacterial communities from BPI, based on their developmental stage and the sex; Figure S9: CAP and PERMANOVA analysis of the bacterial communities of female individuals reared with or without blood meals; Figure S10: The structure of the bacterial communities of wild samples; Figure S11: The presence of the two *Wolbachia* OTUs in the wild and laboratory populations of *Ae*. *albopictus* that were used in the 16S rRNA amplicon survey; Figure S12: Neighbor-joining tree of 16S rRNA sequences of bacterial strains isolated from the larval extract; Table S1: Laboratory populations of *Ae*. *albopictus* used in the survey, including their origin, collection date, and generation; Table S2: Adult individuals from wild populations which were used in the amplicon sequencing analysis of bacterial communities; Table S3: The detailed taxonomic distribution of the filtered reads and the relative abundance of each Operational Taxonomic Unit (OTU); Table S4: Alpha diversity indices of bacterial communities of laboratory and wild populations of *Ae*. *albopictus* samples, Table S5: Alpha diversity indices of bacterial communities of laboratory samples from UTH; Table S6: Alpha diversity indices of bacterial communities of laboratory samples from BPI; Table S7: Alpha diversity indices of bacterial communities of non-blood and blood-reared samples from BPI; Table S8: The presence of the two *Wolbachia* OTUs across all the samples used in the analysis; Table S9: The core bacterial species based on the origin of the population; Table S10: The core bacteria of the laboratory populations based on the developmental stage; Table S11: Isolated bacterial strains from larval and adult extracts grown on three culturing media.
