# Supplementary material for: The Symbiotic Bacterial Profile of Laboratory-Reared and Field-Caught Aedes albopictus Mosquitoes from Greece
Source: Microorganisms. 2025 Jun 26;13(7):1486. doi: 10.3390/microorganisms13071486 (PMC12298842; doi:10.3390/microorganisms13071486)
Supplement: Supplementary file 1 [file microorganisms-13-01486-s001.zip › microorganisms-3658840-supplementary.pdf]

Supplementary Materials

The symbiotic bacterial profile of laboratory reared, and field-caught *Aedes albopictus* mosquitoes from Greece

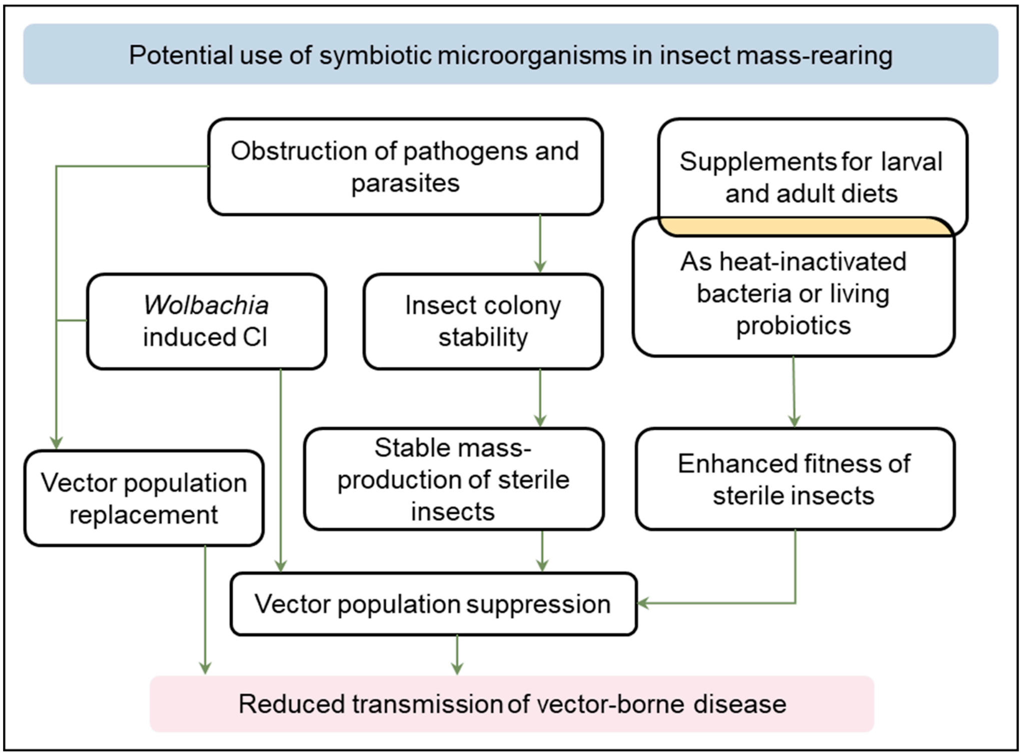

Figure S1. Outline of potential uses of the insect microbiome in mass-rearing and vector-borne disease control.

Table S1. Laboratory populations of *Ae. albopictus* used in the survey, their origin, collection date and generation.

No.	Population	Collection Site	Area	Coordinates	Domestication Date	Generation
1	BPI_P1	Chania	South Greece	35.494248, 24.045501	2021	F_1
2	BPI_P2	Attica	Central Greece	38.081263, 23.812023	2021	F_1
3	BPI_P3	Thessaloniki	North Greece	40.536660, 22.998795	2021	F_1
4	BPI_P4	Kavala	Northeast Greece	40.936135, 24.395156	2021	F_1
5	UTH_P1	Volos	Central Greece	39.374320, 22.945038	2017	F_{20}
6	UTH_P2	Karitsa	Central Greece	39.843543, 22.762999	2021	F_5

Table S2. Adult individuals from wild populations used in the amplicon sequencing analysis of bacterial communities. Species identification was based on amplification and Sanger sequencing of the cytochrome c oxidase subunit I (COI) gene.

Area	Sample code	Sampling period	Coordinates of collection sites	No. of Samples	Samples tested for COI	*Ae. albopictus* positive samples
Vravrona, region of Attica	AT_S1	June 2022	37.919581, 24.011610	20	20	20
	AT_S2	August 2022	37.919581, 24.011610	20	20	20
	AT_S3	October 2022	37.919581, 24.011610	20	20	20
Volos, region of Thessaly	TH_S1	June 2022	39.352928, 22.963126	20	20	20
	TH_S2	August 2022	39.352928, 22.963126	20	20	20
	TH_S3	October 2022	39.362343, 22.951612	20	20	20
**Total no. of samples**				**120**		

Table S3. The detailed taxonomic distribution of the filtered reads and the relative abundance of each Operational Taxonomic Unit (OTU). The two dominant taxa belong to the genus Wolbachia and are highlighted in blue. RA: Relative abundance, SE: Standard error.

	Taxonomy							
**OTU ID**	**Phylum**	**Class**	**Order**	**Family**	**Genus**	**Species**	**RA**	**SE**
Otu80	1. Actinobacteriota	1. Actinobacteria	1. Micrococcales	1. Microbacteriaceae	1. Leifsonia	1. Microbacteriaceae_bacterium	0.21	0.06
Otu8					2. Leucobacter	2. Leucobacter_iarius	0.29	0.1
Otu6					3. Microbacterium	3. uncultured_Microbacterium	5.16	0.89
Otu28			2. Propionibacteriales	2. Propionibacteriaceae	4. Cutibacterium	4. uncultured_Cutibacterium	0.25	0.04
Otu106	2. Bacteroidota	2. Bacteroidia	3. Bacteroidales	3. Dysgonomonadaceae	5. unassigned	5. unassigned	0.16	0.13
Otu15			4. Flavobacteriales	4. Weeksellaceae	6. Chryseobacterium	6. Chryseobacterium_sp.	0.59	0.2
Otu194					7. Elizabethkingia	7. Elizabethkingia_sp.1	0.22	0.02
Otu228						8. Elizabethkingia_sp.2	0.12	0.01
Otu541						9. Elizabethkingia_sp.3	0.50	0.05
Otu2						10. Elizabethkingia_ursingii	10.30	0.98
Otu14			5. Sphingobacteriales	5. Sphingobacteriaceae	8. Sphingobacterium	11. Sphingobacterium_sp.	0.12	0.03
Otu35	3. Firmicutes	3. Bacilli	6. Bacillales	6. Bacillaceae	9. Bacillus	12. Bacillus_anthracis	0.11	0.06
Otu61					10. Geobacillus	13. Geobacillus_stearothermophilus	0.26	0.07
Otu41			7. Brevibacillales	7. Brevibacillaceae	11. Brevibacillus	14. Brevibacillus_agri	0.14	0.03
Otu25			8. Lactobacillales	8. Leuconostocaceae	12. Fructobacillus	15. Fructobacillus_tropaeoli	0.16	0.16
Otu5	4. Proteobacteria	4. Alphaproteobacteria	9. Acetobacterales	9. Acetobacteraceae	13. Asaia	16. Asaia_siamensis	4.06	0.45
Otu31			10. Caulobacterales	10. Caulobacteraceae	14. Brevundimonas	17. Brevundimonas_vesicularis	0.59	0.17
Otu11			11. Rhizobiales	11. Beijerinckiaceae	15. Bosea	18. uncultured_Bosea	0.72	0.12
Otu36				12. Rhizobiaceae	16. Allorhizobium-Neorhizobium-Pararhizobium-Rhizobium	19. Rhizobium_sp.	0.14	0.04
Otu12					17. Phyllobacterium	20. uncultured_Phyllobacterium	0.20	0.03
Otu19					18. Shinella	21. Zoogloea_ramigera	0.28	0.05
Otu18				13. Xanthobacteraceae	19. Bradyrhizobium	22. uncultured_Bradyrhizobium	0.12	0.03
Otu1			12. Rickettsiales	14. Anaplasmataceae	20. Wolbachia	23. Wolbachia_endosymbiont	42.23	1.4
Otu3						24. Wolbachia_of_Calligrapha_confluens	21.50	0.98
Otu17			13. Sphingomonadales	15. Sphingomonadaceae	21. Sphingobium	25. Sphingobium_sp.1	0.33	0.1
Otu23						26. Sphingobium_sp.2	0.67	0.17
Otu47					22. Sphingomonas	27. Sphingomonas_koreensis	0.25	0.06
Otu22						28. Sphingomonas_sp.	0.12	0.03

Otu30		5. Gammaproteobacteria	14. Aeromonadales	16. Aeromonadaceae	23. Aeromonas	29. uncultured_Aeromonas	0.24	0.04
Otu38			15. Burkholderiales	17. Comamonadaceae	24. Delftia	30. Delftia_acidovorans	0.24	0.06
Otu16				18. Methylophilaceae	25. Methylophilus	31. uncultured_Methylophilus	0.28	0.11
Otu32				19. Neisseriaceae	26. Neisseria	32. uncultured_Neisseria_1	0.49	0.16
Otu144						33. uncultured_Neisseria_2	0.26	0.09
Otu9			16. Enterobacterales	20. Erwiniaceae	27. Pantoea	34. Pantoea_agglomerans	1.66	0.54
Otu4				21. Yersiniaceae	28. Serratia	35. Serratia_marcescens	2.69	0.35
Otu7			17. Oceanospirillales	22. Halomonadaceae	29. Zymobacter	36. uncultured_Zymobacter	1.85	0.56
Otu69			18. Pasteurellales	23. Pasteurellaceae	30. Haemophilus	37. uncultured_Haemophilus	0.31	0.08
Otu13			19. Pseudomonadales	24. Moraxellaceae	31. Acinetobacter	38. Acinetobacter_johnsonii	0.54	0.1
Otu27						39. uncultured_Acinetobacter	0.21	0.16
Otu48					32. Enhydrobacter	40. Enhydrobacter_sp.	0.12	0.03
Otu45				25. Pseudomonadaceae	33. Pseudomonas	41. Pseudomonas_resinovorans	0.17	0.04
Otu10						42. Pseudomonas_sp.	0.84	0.1
Otu42			20. Vibrionales	26. Vibrionaceae	34. Vibrio	43. Vibrio_metschnikovii	0.32	0.08

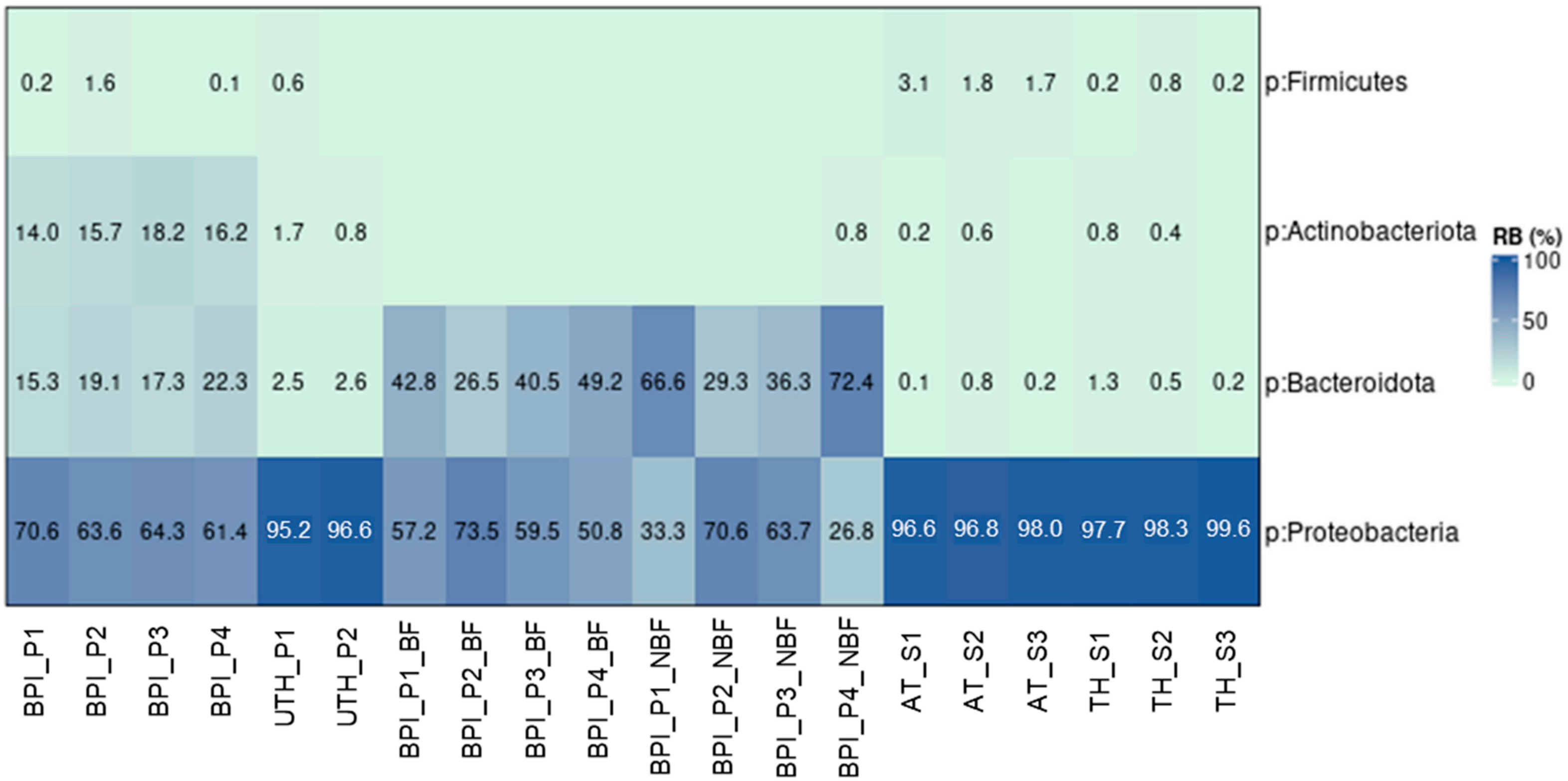

Figure S2. **The bacterial composition at the phylum level (p) of all the sample groups used in the 16S rRNA amplicon survey**. BPI (lab population from Attica), UTH (lab population from Thessaly), BF (blood-fed females from Attica), NBF (non-blood fed females from Attica), AT (field-caught samples from Attica), TH (field-caught samples from Thessaly).

Table S4. Alpha diversity indices of bacterial communities of laboratory and wild populations of *Ae. albopictus* samples. Statistically significant differences (ANOVA, p≤0.05) are described with different letters.

	ACE	Chao1	Shannon	Simpson
**Lab**	16.56±0.34[a]	17.35±0.4[a]	1.29±0.03[a]	0.6±0.01[a]
**Wild**	11.1±0.3[b]	12.43±0.41[b]	0.75±0.02[b]	0.44±0.01[b]

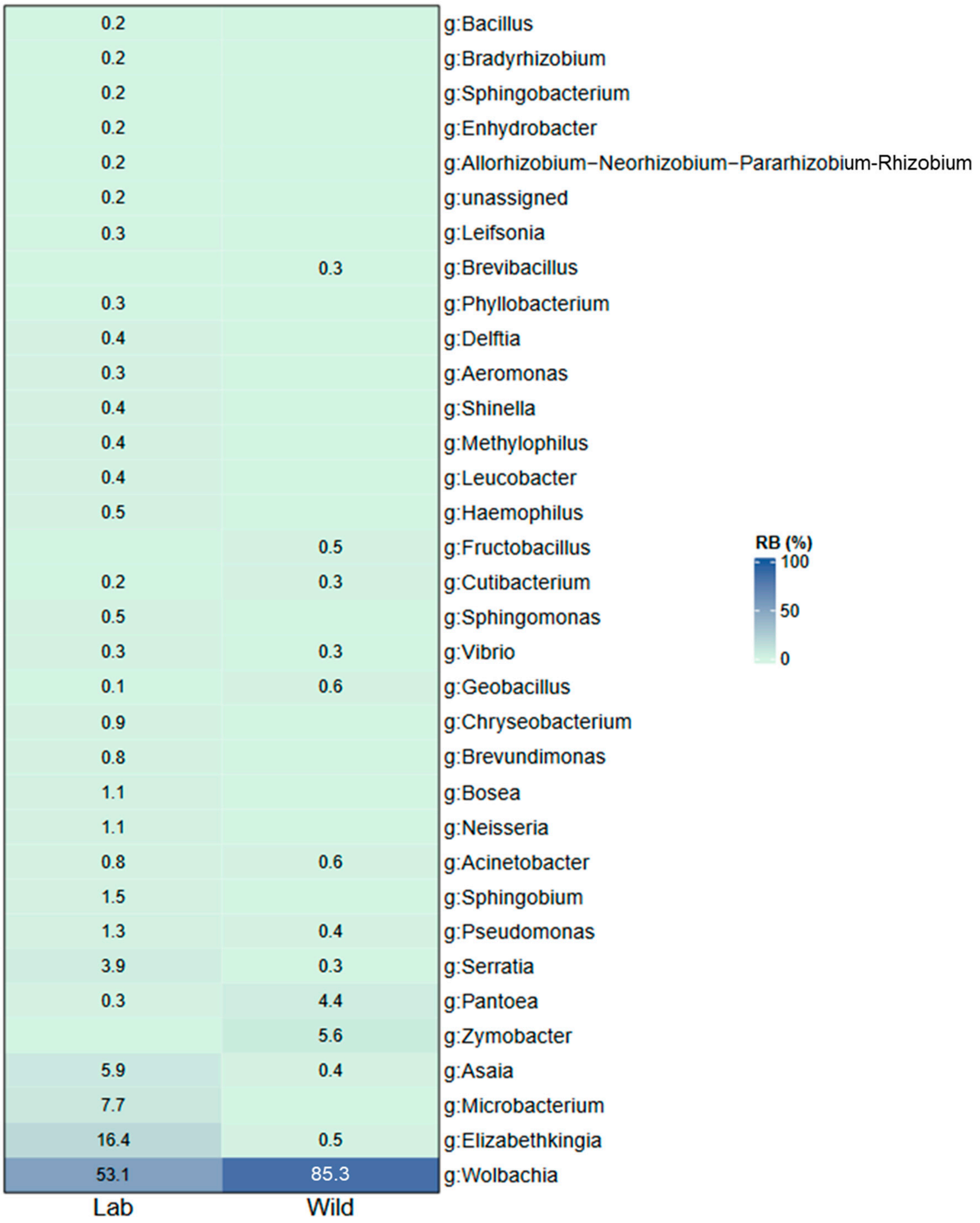

Figure S3. The bacterial communities at the genus level (g) of laboratory reared and wild populations of *Ae. albopictus* mosquitoes.

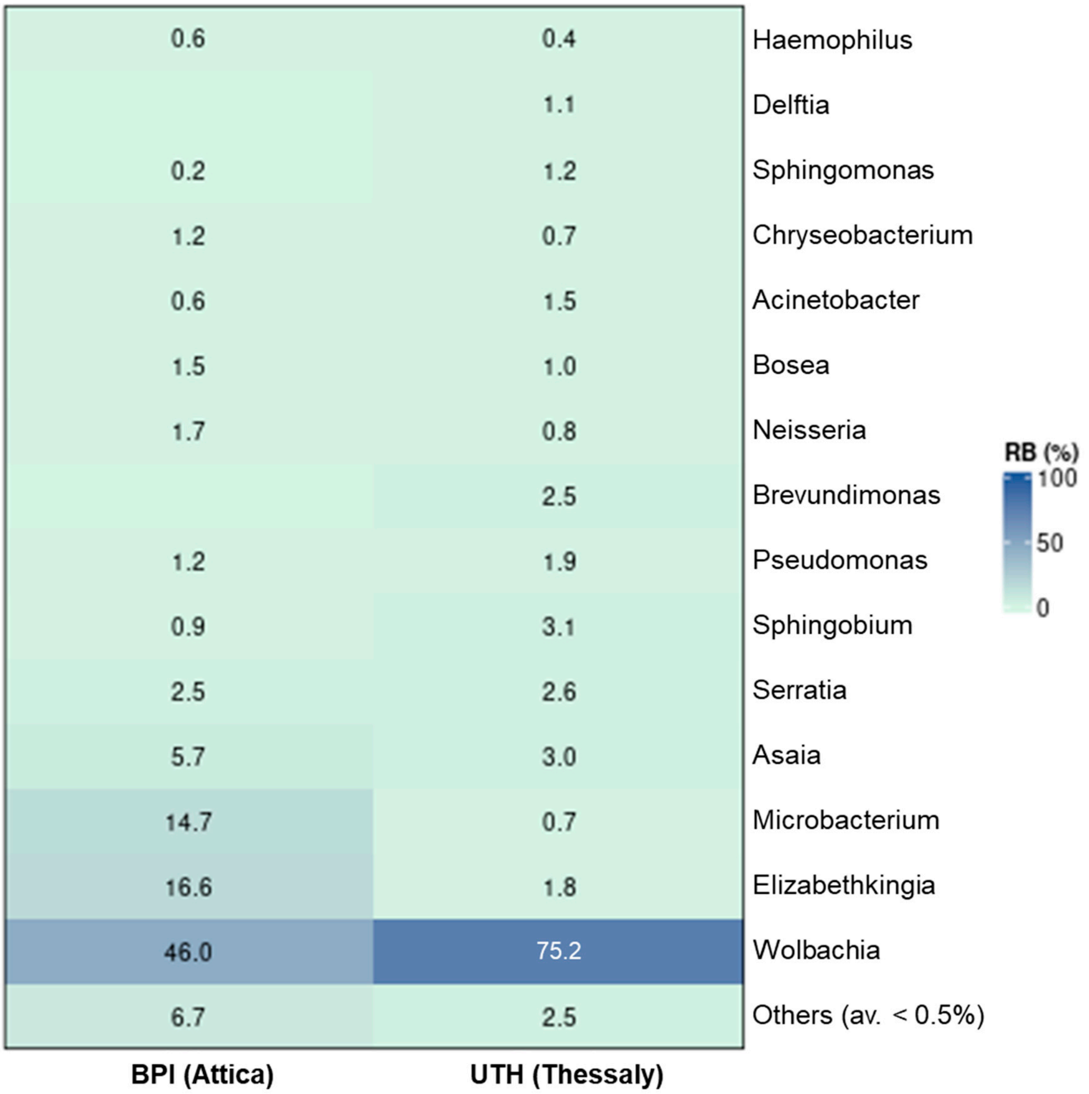

Figure S4. Relative abundance of bacterial genera in the laboratory populations of BPI and UTH.

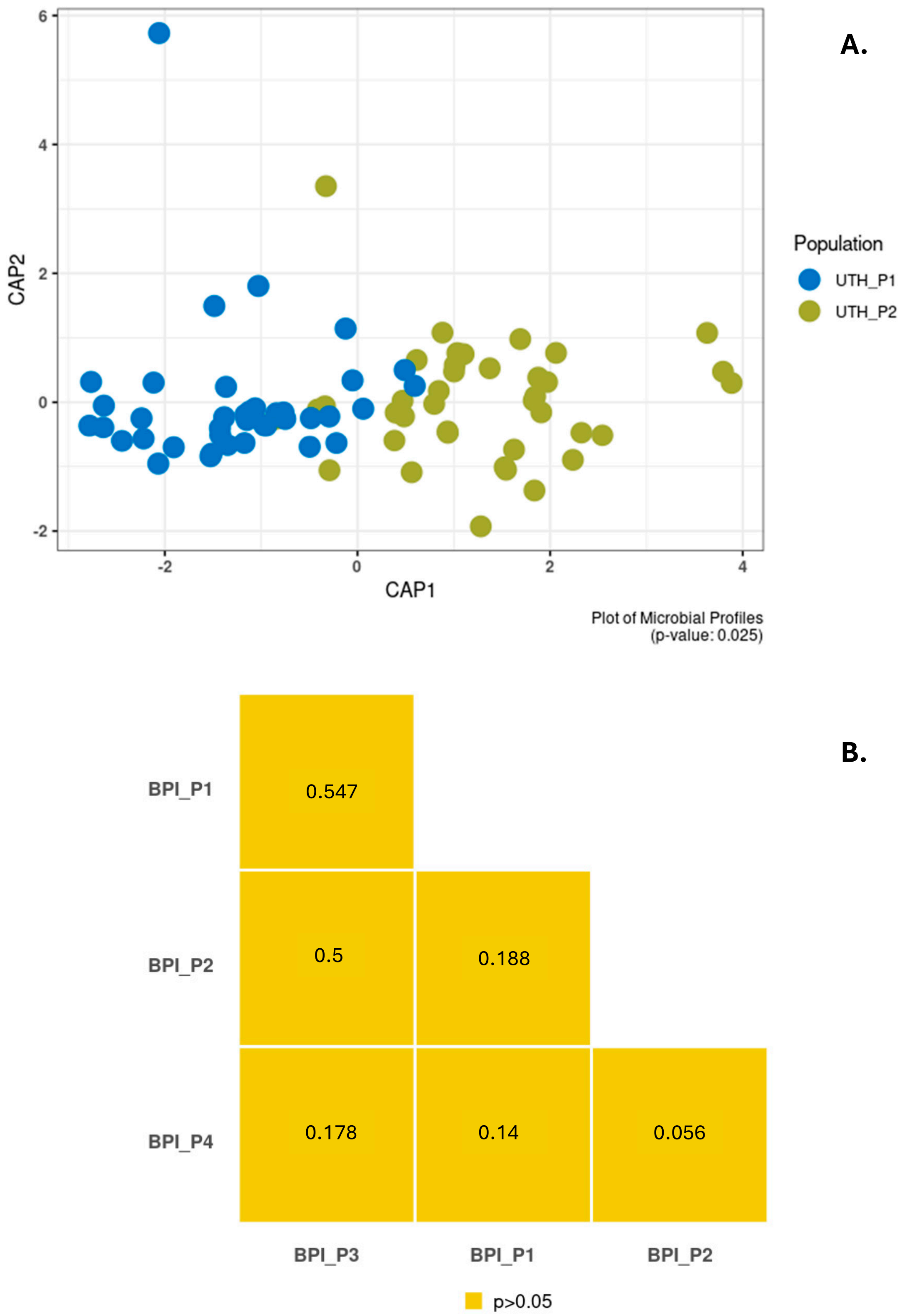

Figure S5. The structure of the bacterial communities of lab populations. (A) UTH lab populations developed distinct bacterial communities (PERMANOVA, p=0.025; betadisper, p≤0.05). (B) BPI populations formed similar bacteriomes (PERMANOVA, p>0.05).

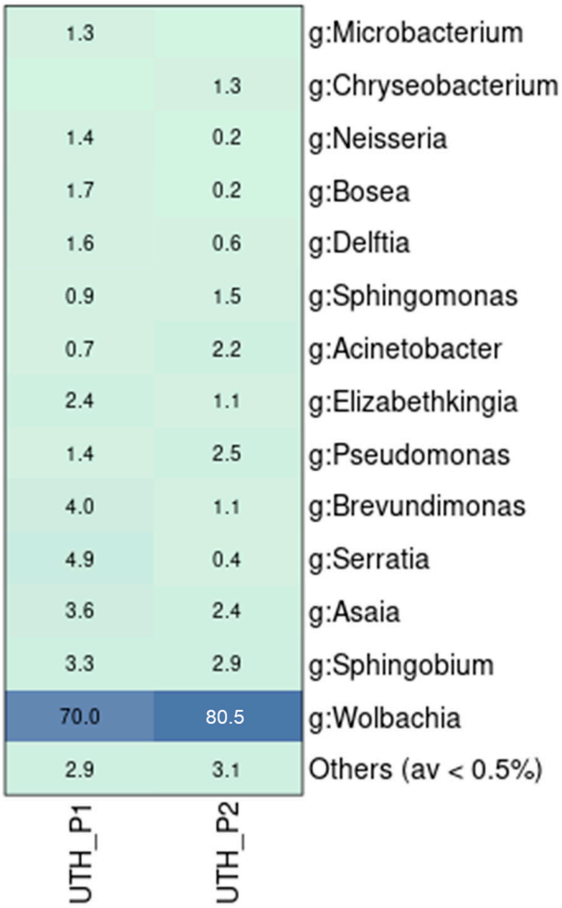

Figure S6. Relative abundance of bacterial genera in the laboratory populations from UTH. The two populations displayed differences in all identified genera. Other bacterial genera with less than 0.5% average relative abundance (Others) were grouped.

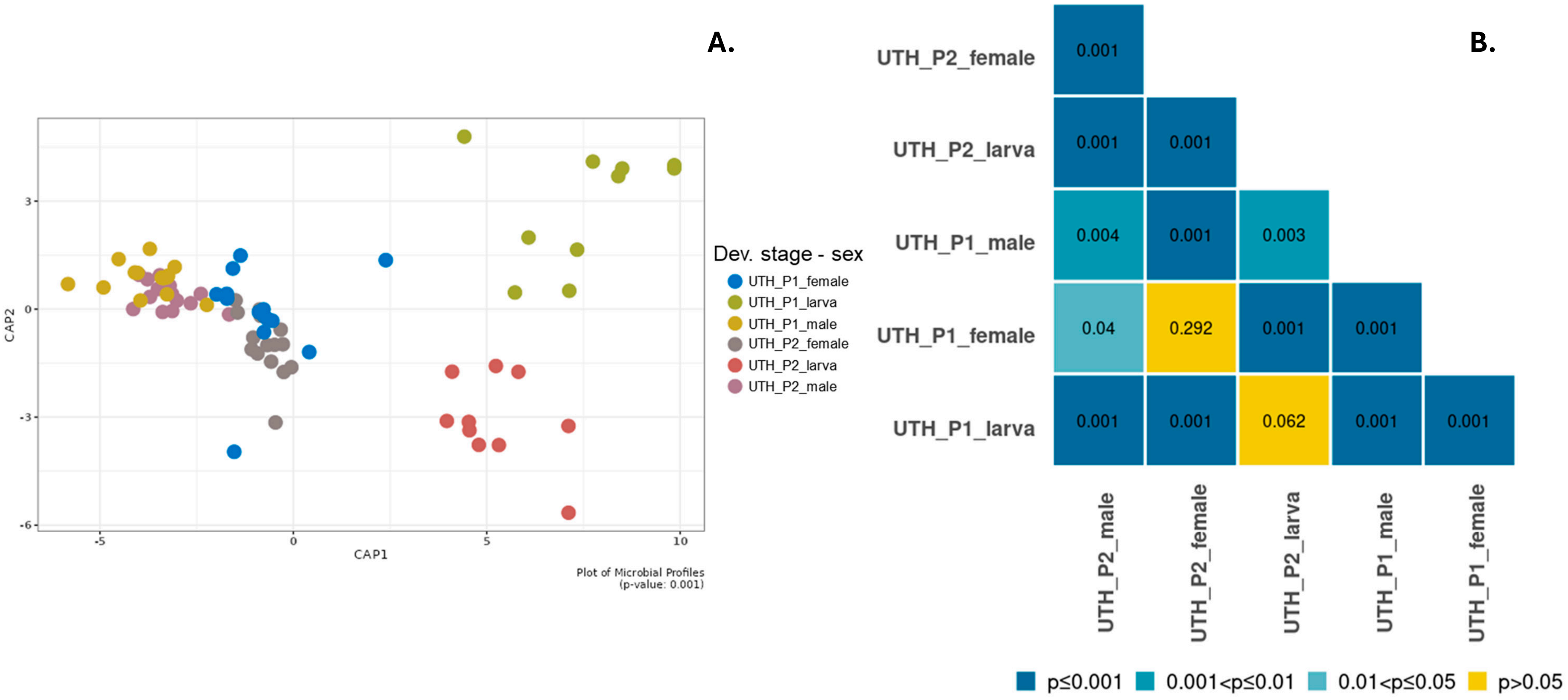

Figure S7. Community structure of UTH laboratory populations according to the developmental stage and the sex of the flies. (A) CAP plot displaying sample clustering. (B) Table with pairwise comparison of PERMANOVA p-values. Statistically significant differences are highlighted in blue (PERMANOVA, $p \leq 0.05$). UTH_P1 females shared similar bacterial profiles with UTH_P2 females. The same was the case for UTH_P1 and UTH_P2 larvae. In three pairwise comparisons, "UTH_P1_male vs UTH_P1_female", "UTH_P1_male vs UTH_P2_male", and "UTH_P1_male vs UTH_P2_female", statistically different dispersions were observed (PERMANOVA, $p \leq 0.05$; betadisper, $p \leq 0.05$).

Table S5. Alpha diversity indices of bacterial communities of laboratory samples from UTH. Statistically significant differences between groups (ANOVA, p≤0.05) are described with different letters, and similarities with similar letters.

UTH	ACE	Chao1	Shannon	Simpson
**Female**	14.57±0.66^{c}	16.67±1.2^{c}	0.93±0.04^{b}	0.53±0.01^{b}
**Larva**	22.17±0.64^{a}	22.55±0.74^{a}	2.07±0.07^{a}	0.8±0.02^{a}
**Male**	19.2±0.69^{b}	20.91±1.07^{a,b}	1.05±0.12^{b}	0.45±0.05^{b}

Table S6. Alpha diversity indices of bacterial communities of laboratory samples from BPI. Statistically significant differences between groups (ANOVA, p≤0.05) are described with different letters.

BPI	ACE	Chao1	Shannon	Simpson
**Female**	15.7±0.88^{a}	15.55±0.66^{c}	1.38±0.05^{b}	0.66±0.02^{c}
**Larva**	17.72±0.73^{a}	19.48±1.18^{a}	1.18±0.05^{a}	0.53±0.02^{a}
**Male**	16.95±0.66^{a}	17.72±0.75^{a,b}	1.23±0.06^{a,b}	0.56±0.02^{a,b}

Table S7. Alpha diversity indices of bacterial communities of non-blood and blood reared samples from BPI. Statistically significant differences between groups (ANOVA, p≤0.05) are described with different letters.

BPI blood-fed	ACE	Chao1	Shannon	Simpson
**Blood-fed**	12.3±1.1^{a}	12.08±0.84^{a}	1.48±0.04^{a}	0.72±0.01^{a}
**Non-blood-fed**	13.39±1.01^{a}	13.05±0.76^{a}	1.35±0.06^{a}	0.64±0.03^{b}

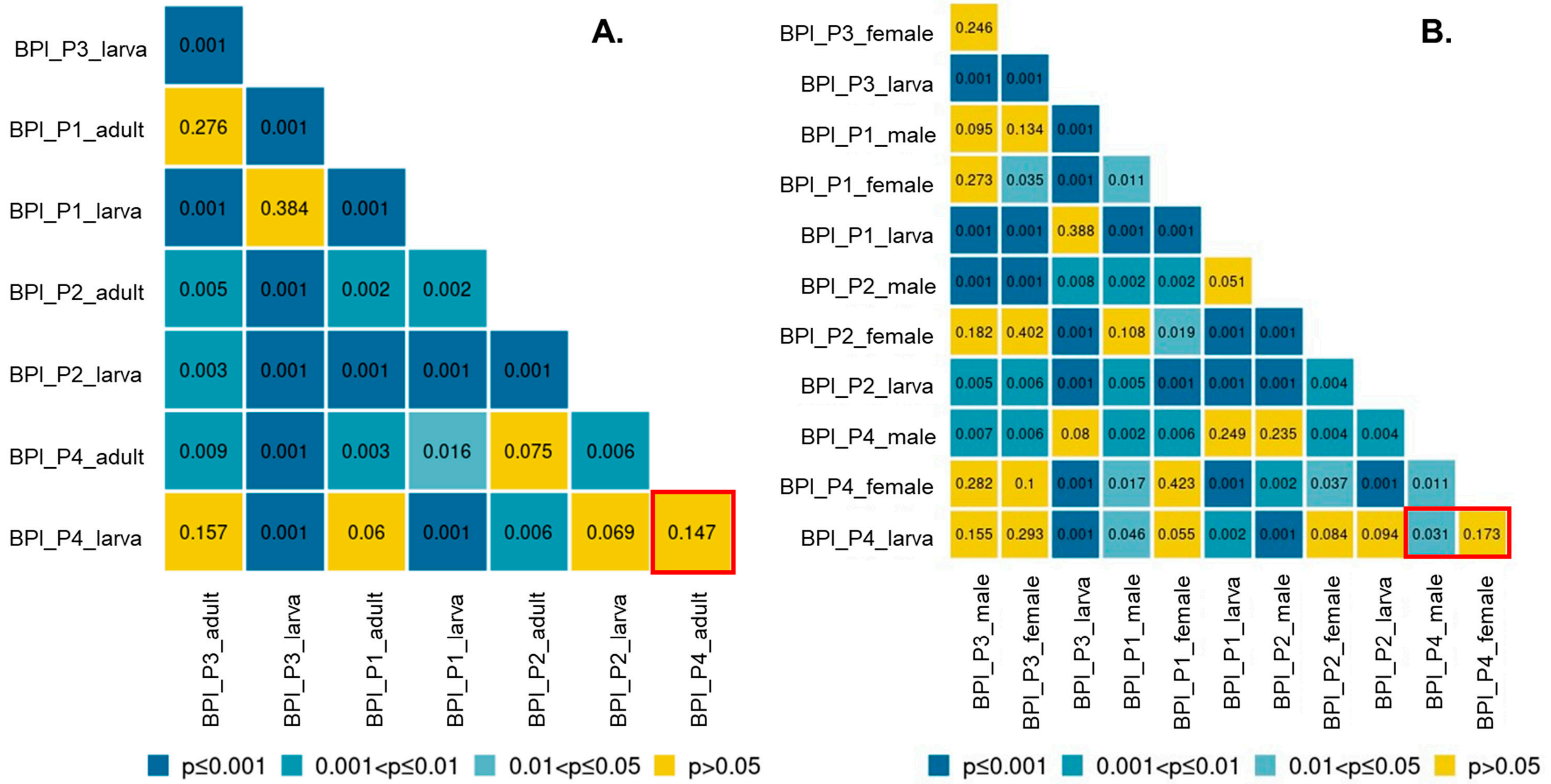

Figure S8. Pairwise comparison of mosquito bacterial communities from BPI, based on their developmental stage and the sex. PERMANOVA p-values are presented on the tables. Significant differences are highlighted in blue (p≤0.05). Red rectangles highlight the comparison between larvae and adults from P4. In one comparison, “BPI_P3_larva vs BPI_P4_male”, statistically different dispersions were observed between the groups (PERMANOVA, p>0.05; betadisper p≤0.05).

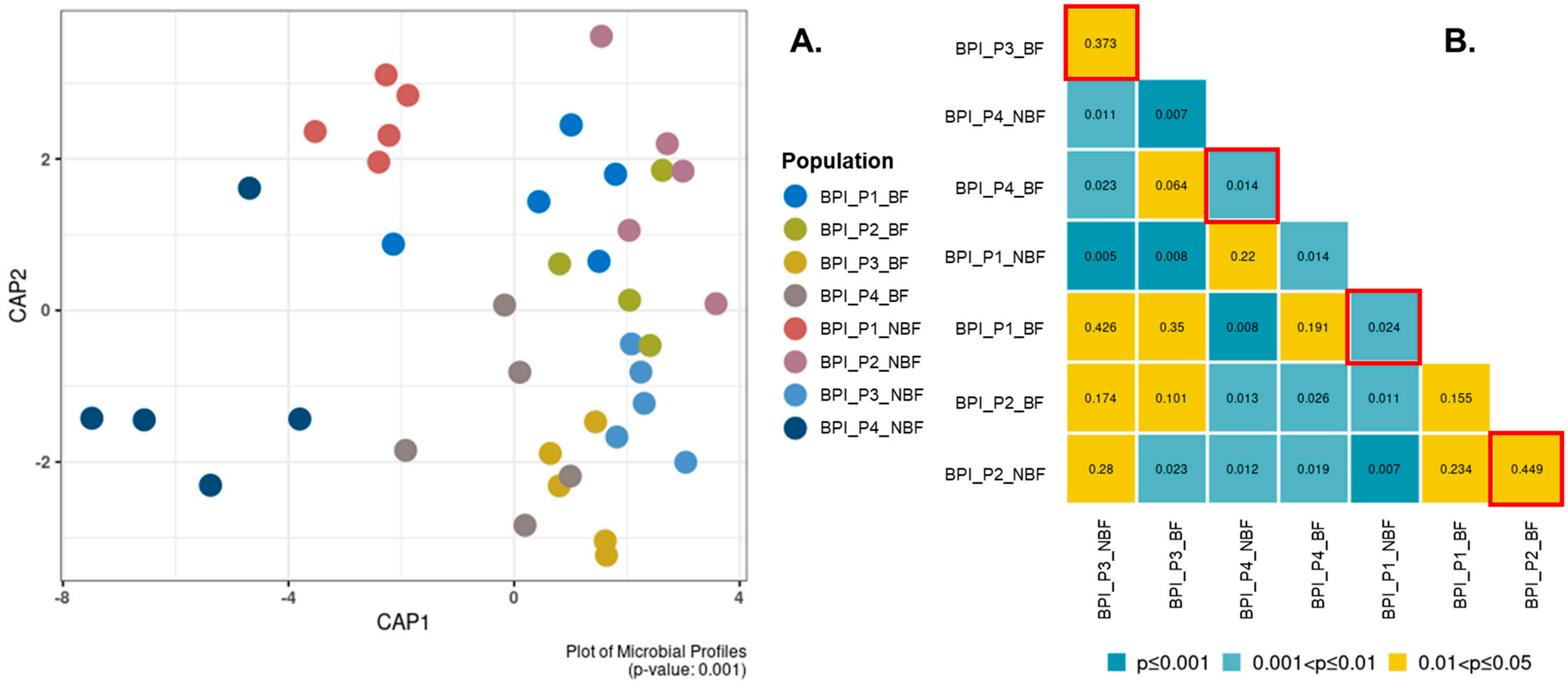

Figure S9. CAP and PERMANOVA analysis of the bacterial communities of female individuals reared with or without blood meals. The populations were reared in the facilities of BPI, in Attica. Statistically significant differences are highlighted in blue (p≤0.05).

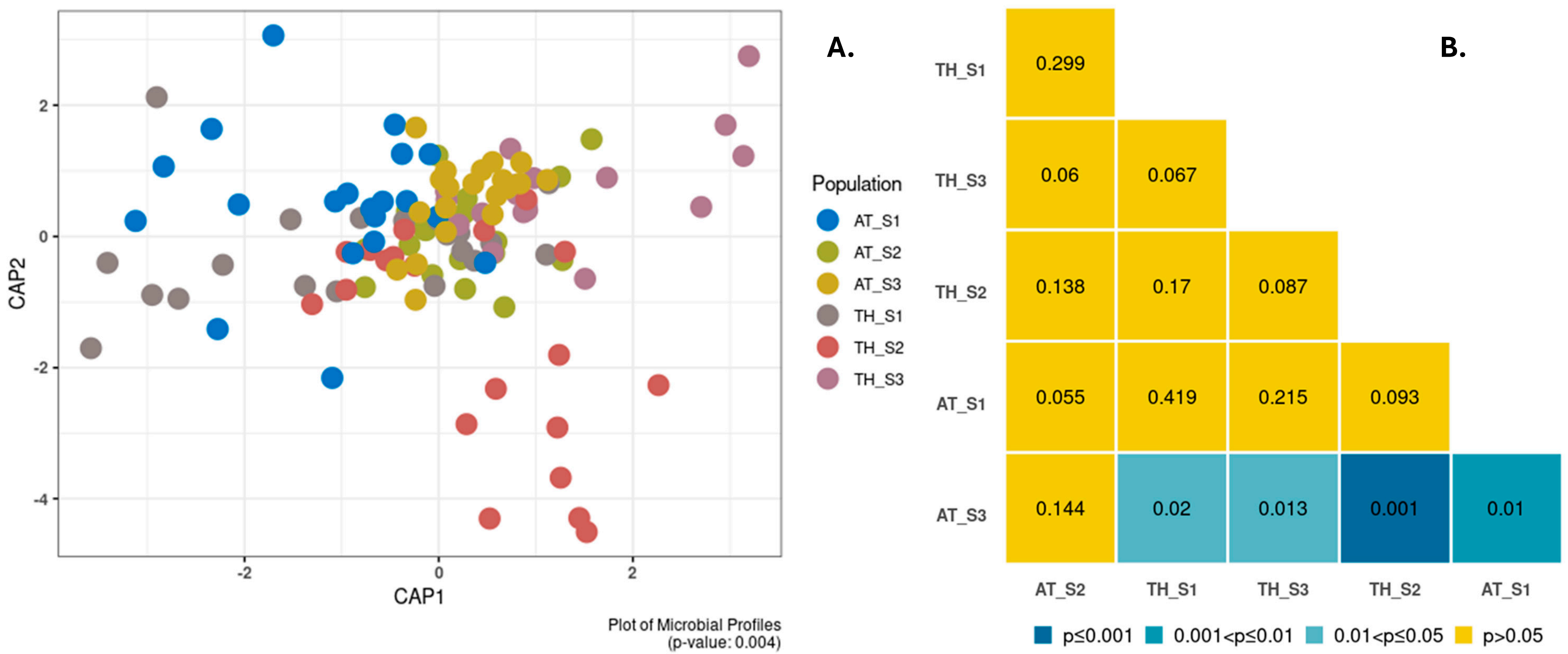

Figure S10. The structure of the bacterial communities of wild samples. (A) CAP plot and (B) PERMANOVA table of bacterial communities identified in wild Ae. albopictus samples. Sampling was performed in Attica (AT) and Thessaly (TH) in three different time periods: S1: June, S2: August, and S3: October. Significant differences are highlighted in blue (p≤0.05).

Wild populations

	Attica (AT)	Thessaly (TH)
Otu3 - wAlbA	33.19	27.16
Otu1 - wAlbB	55.09	55.09

Permanova comparison of *Wolbachia* between AT and BPI: p-value = 0.007

Permanova comparison of *Wolbachia* between TH and UTH: p-value = 0.184

	Attica June (AT_S1)	Attica August (AT_S2)	Attica October (AT_S3)	Thessaly June (TH_S1)	Thessaly August (TH_S2)	Thessaly October (TH_S3)
Otu3 - wAlbA	36.17	30.00	33.40	31.37	28.36	21.75
Otu1 -wAlbB	43.05	59.33	62.88	54.32	54.23	56.74

Laboratory populations

	BPI (AT)	UTH (Thessaly)
Otu3 - wAlbA	12.96	25.89
Otu1 - wAlbB	33.03	49.33

	BPI_P1	BPI_P2	BPI_P3	BPI_P4	UTH_P1	UTH_P2
Otu3 - wAlbA	13.61	13.32	9.09	15.82	25.72	26.07
Otu1 - wAlbB	35.97	22.15	36.60	37.40	44.25	54.41

	BPI_P1_NBF	BPI_P1_BF	BPI_P2_NBF	BPI_P2_BF	BPI_P3_NBF	BPI_P3_BF	BPI_P4_NBF	BPI_P4_BF
Otu3 - wAlbA	19.15	13.24	18.58	20.04	9.08	7.86	7.31	10.14
Otu1 - wAlbB	12.66	13.14	13.50	27.21	20.21	15.51	16.71	21.63

Figure S11. The presence of the two *Wolbachia* OTUs in the wild and laboratory populations of *Ae. albopictus* that were used in the 16S rRNA amplicon survey.

Table S8. The presence of the two *Wolbachia* OTUs across all the samples used in the analysis. The table was sorted based on the second column (No. of reads OTU3 - *w*AlbA, highlighted in grey), from the largest to the smallest value.

Sample ID	No. of reads OTU3 *w*AlbA	No. of reads OTU1 -*w*AlbB	OTU3 - *w*AlbA %	OTU1 - *w*AlbB %	Total reads	Population	Developmental stage - Sex	Feed	Origin	Location	Sampling
AT_S1_41	3469	1474	69.93	29.71	4961	AT_S1	Adult	Sugar	Wild	Attica	June
TH_S2 _1	3386	1423	69.29	29.12	4887	TH_S2	Adult	Sugar	Wild	Thessaly	August
AT_S1_27	3243	1589	65.32	32.00	4965	AT_S1	Adult	Sugar	Wild	Attica	June
AT_S1_23	3186	1778	63.89	35.65	4987	AT_S1	Adult	Sugar	Wild	Attica	June
TH_S1_27	3141	1741	63.97	35.46	4910	TH_S1	Adult	Sugar	Wild	Thessaly	June
TH_S1_4	3085	1832	61.99	36.81	4977	TH_S1	Adult	Sugar	Wild	Thessaly	June
TH_S2 _2	2997	1929	60.40	38.88	4962	TH_S2	Adult	Sugar	Wild	Thessaly	August
AT_S1_25	2959	1647	62.15	34.59	4761	AT_S1	Adult	Sugar	Wild	Attica	June
TH_S2_24	2892	1994	58.40	40.27	4952	TH_S2	Adult	Sugar	Wild	Thessaly	August
AT_S2_9	2887	2022	57.90	40.55	4986	AT_S2	Adult	Sugar	Wild	Attica	October
UTH_P2_14D_F_C	2880	2008	58.11	40.52	4956	UTH_P2	Female	Sugar	Lab	Thessaly	-
AT_S1_39	2879	2101	57.66	42.08	4993	AT_S1	Adult	Sugar	Wild	Attica	June
AT_S3_34	2809	1981	56.60	39.92	4963	AT_S3	Adult	Sugar	Wild	Attica	August
BPI_P4_14dM_D	2784	1847	57.32	38.03	4857	BPI_P4	Male	Sugar	Lab	Attica	-
UTH_P1_1dF_E	2760	2168	55.51	43.60	4972	UTH_P1	Female	Sugar	Lab	Thessaly	-
AT_S1_34	2742	2141	56.09	43.79	4889	AT_S1	Adult	Sugar	Wild	Attica	June
UTH_P1_L3_E	2725	748	60.87	16.71	4477	UTH_P1	Larva	Sugar	Lab	Thessaly	-
AT_S2_27	2725	2232	54.68	44.78	4984	AT_S2	Adult	Sugar	Wild	Attica	October
TH_S2_22	2674	2094	55.17	43.20	4847	TH_S2	Adult	Sugar	Wild	Thessaly	August
UTH_P1_14dF_D	2661	2275	53.57	45.80	4967	UTH_P1	Female	Sugar	Lab	Thessaly	-
AT_S3_22	2646	1862	54.51	38.36	4854	AT_S3	Adult	Sugar	Wild	Attica	August
AT_S1_33	2631	2244	53.76	45.85	4894	AT_S1	Adult	Sugar	Wild	Attica	June
BPI_P1_3dF_E	2595	2244	52.59	45.48	4934	BPI_P1	Female	Sugar	Lab	Attica	-
AT_S1_31	2594	2281	52.96	46.57	4898	AT_S1	Adult	Sugar	Wild	Attica	June
UTH_P2_14D_F_A	2562	2284	51.78	46.16	4948	UTH_P2	Female	Sugar	Lab	Thessaly	-
AT_S1_29	2538	2424	50.93	48.65	4983	AT_S1	Adult	Sugar	Wild	Attica	June
AT_S3_40	2533	986	57.45	22.36	4409	AT_S3	Adult	Sugar	Wild	Attica	August
BPI_P2_14dM_B	2528	1297	55.32	28.38	4570	BPI_P2	Male	Sugar	Lab	Attica	-
BPI_P1_3dF_B	2523	1422	52.51	29.59	4805	BPI_P1	Female	Sugar	Lab	Attica	-
TH_S1_33	2520	2426	50.54	48.66	4986	TH_S1	Adult	Sugar	Wild	Thessaly	June
UTH_P1_14dF_E	2519	2330	51.00	47.18	4939	UTH_P1	Female	Sugar	Lab	Thessaly	-
UTH_P1_14dF_B	2505	2419	50.35	48.62	4975	UTH_P1	Female	Sugar	Lab	Thessaly	-

UTH_P2_14D_F_E	2501	2396	50.60	48.47	4943	UTH_P2	Female	Sugar	Lab	Thessaly	-
AT_S1_24	2498	2481	50.05	49.71	4991	AT_S1	Adult	Sugar	Wild	Attica	June
BPI_P4_14dM_E	2496	2269	51.14	46.49	4881	BPI_P4	Male	Sugar	Lab	Attica	-
AT_S3_41	2456	2474	49.54	49.90	4958	AT_S3	Adult	Sugar	Wild	Attica	August
AT_S3_39	2427	2427	48.96	48.96	4957	AT_S3	Adult	Sugar	Wild	Attica	August
UTH_P2_L3_B	2420	1466	53.46	32.38	4527	UTH_P2	Larva	Sugar	Lab	Thessaly	-
AT_S3_35	2413	2239	49.42	45.85	4883	AT_S3	Adult	Sugar	Wild	Attica	August
TH_S3_4	2412	2349	48.73	47.46	4950	TH_S3	Adult	Sugar	Wild	Thessaly	October
UTH_P1_14dF_C	2406	2507	48.42	50.45	4969	UTH_P1	Female	Sugar	Lab	Thessaly	-
UTH_P1_1dF_D	2361	2523	47.77	51.05	4942	UTH_P1	Female	Sugar	Lab	Thessaly	-
UTH_P2_3D_F_A	2348	2407	48.41	49.63	4850	UTH_P2	Female	Sugar	Lab	Thessaly	-
AT_S2_33	2347	2548	47.01	51.03	4993	AT_S2	Adult	Sugar	Wild	Attica	October
AT_S2_25	2277	2668	45.87	53.75	4964	AT_S2	Adult	Sugar	Wild	Attica	October
BPI_P3_3d_F_C	2271	2012	46.53	41.22	4881	BPI_P3	Female	Sugar	Lab	Attica	-
TH_S1_16	2269	2685	45.61	53.97	4975	TH_S1	Adult	Sugar	Wild	Thessaly	June
AT_S2_31	2266	2575	45.60	51.82	4969	AT_S2	Adult	Sugar	Wild	Attica	October
TH_S1_41	2250	1292	45.76	26.28	4917	TH_S1	Adult	Sugar	Wild	Thessaly	June
AT_S1_40	2250	2684	45.38	54.14	4958	AT_S1	Adult	Sugar	Wild	Attica	June
UTH_P2_1D_F_C	2229	2352	45.57	48.09	4891	UTH_P2	Female	Sugar	Lab	Thessaly	-
BPI_P4_14dM_C	2225	2658	44.98	53.73	4947	BPI_P4	Male	Sugar	Lab	Attica	-
TH_S3_50	2223	2449	47.25	52.05	4705	TH_S3	Adult	Sugar	Wild	Thessaly	October
TH_S1_34	2215	1863	45.78	38.51	4838	TH_S1	Adult	Sugar	Wild	Thessaly	June
TH_S3_32	2208	2736	44.41	55.03	4972	TH_S3	Adult	Sugar	Wild	Thessaly	October
UTH_P2_3D_F_D	2203	2443	45.34	50.28	4859	UTH_P2	Female	Sugar	Lab	Thessaly	-
UTH_P2_L3_D	2197	1364	51.90	32.22	4233	UTH_P2	Larva	Sugar	Lab	Thessaly	-
TH_S2_10	2186	2700	44.49	54.95	4914	TH_S2	Adult	Sugar	Wild	Thessaly	August
UTH_P2_14D_F_B	2185	2650	44.28	53.70	4935	UTH_P2	Female	Sugar	Lab	Thessaly	-
AT_S3_33	2184	2632	44.19	53.26	4942	AT_S3	Adult	Sugar	Wild	Attica	August
AT_S2_15	2158	2256	43.31	45.27	4983	AT_S2	Adult	Sugar	Wild	Attica	October
AT_S1_35	2155	2808	43.22	56.32	4986	AT_S1	Adult	Sugar	Wild	Attica	June
BPI_P2_14dM_E	2153	1902	45.83	40.49	4698	BPI_P2	Male	Sugar	Lab	Attica	-
AT_S2_39	2143	2214	46.17	47.70	4642	AT_S2	Adult	Sugar	Wild	Attica	October
TH_S1_5	2086	2084	42.84	42.80	4869	TH_S1	Adult	Sugar	Wild	Thessaly	June
BPI_P3_1d_F_B	2054	2774	41.33	55.82	4970	BPI_P3	Female	Sugar	Lab	Attica	-
TH_S1_6	2048	2088	42.06	42.88	4869	TH_S1	Adult	Sugar	Wild	Thessaly	June
UTH_P1_1dF_C	1998	2539	41.26	52.44	4842	UTH_P1	Female	Sugar	Lab	Thessaly	-
UTH_P2_3D_F_E	1994	2234	41.29	46.26	4829	UTH_P2	Female	Sugar	Lab	Thessaly	-

TH_S1_8	1985	2904	39.86	58.31	4980	TH_S1	Adult	Sugar	Wild	Thessaly	June
AT_S2_21	1955	2979	39.37	59.99	4966	AT_S2	Adult	Sugar	Wild	Attica	October
UTH_P2_1D_F_E	1951	2250	42.11	48.57	4633	UTH_P2	Female	Sugar	Lab	Thessaly	-
AT_S2_3	1947	2973	39.37	60.11	4946	AT_S2	Adult	Sugar	Wild	Attica	October
TH_S3_21	1887	3055	37.99	61.51	4967	TH_S3	Adult	Sugar	Wild	Thessaly	October
UTH_P1_1dF_B	1870	1679	42.25	37.94	4426	UTH_P1	Female	Sugar	Lab	Thessaly	-
AT_S3_29	1865	3110	37.36	62.30	4992	AT_S3	Adult	Sugar	Wild	Attica	August
TH_S2_26	1863	2659	40.86	58.32	4559	TH_S2	Adult	Sugar	Wild	Thessaly	August
UTH_P2_3D_F_C	1827	2951	37.32	60.27	4896	UTH_P2	Female	Sugar	Lab	Thessaly	-
UTH_P2_14D_F_D	1824	3049	36.60	61.19	4983	UTH_P2	Female	Sugar	Lab	Thessaly	-
BPI_P2_1dF_B	1816	1675	39.22	36.18	4630	BPI_P2	Female	Sugar	Lab	Attica	-
TH_S1_30	1809	3133	36.40	63.04	4970	TH_S1	Adult	Sugar	Wild	Thessaly	June
BPI_P1_1dF_C	1787	2874	36.87	59.29	4847	BPI_P1	Female	Sugar	Lab	Attica	-
BPI_P1_3dF_D	1786	2715	36.99	56.22	4829	BPI_P1	Female	Sugar	Lab	Attica	-
AT_S3_43	1784	3092	36.51	63.28	4886	AT_S3	Adult	Sugar	Wild	Attica	August
TH_S3_23	1783	2546	41.09	58.68	4339	TH_S3	Adult	Sugar	Wild	Thessaly	October
UTH_P2_1D_F_D	1768	2936	36.27	60.23	4875	UTH_P2	Female	Sugar	Lab	Thessaly	-
UTH_P1_3dF_A	1761	3166	35.50	63.82	4961	UTH_P1	Female	Sugar	Lab	Thessaly	-
TH_S1_14	1749	3224	35.07	64.65	4987	TH_S1	Adult	Sugar	Wild	Thessaly	June
AT_S1_47	1745	2027	37.18	43.18	4694	AT_S1	Adult	Sugar	Wild	Attica	June
UTH_P1_3dF_E	1723	2225	35.79	46.22	4814	UTH_P1	Female	Sugar	Lab	Thessaly	-
UTH_P2_1D_M_A	1717	2779	35.91	58.11	4782	UTH_P2	Male	Sugar	Lab	Thessaly	-
AT_S3_25	1705	2844	34.77	57.99	4904	AT_S3	Adult	Sugar	Wild	Attica	August
UTH_P2_1D_F_A	1695	2874	34.88	59.14	4860	UTH_P2	Female	Sugar	Lab	Thessaly	-
UTH_P1_3dF_C	1680	3102	34.22	63.18	4910	UTH_P1	Female	Sugar	Lab	Thessaly	-
P2_BPI_14dF_BF_D	1671	2167	33.77	43.80	4948	BPI_P2_BF	Female	Blood	Lab	Attica	-
UTH_P1_L3_D	1661	1248	38.43	28.88	4322	UTH_P1	Larva	Sugar	Lab	Thessaly	-
AT_S3_17	1658	3312	33.30	66.52	4979	AT_S3	Adult	Sugar	Wild	Attica	August
BPI_P4_1dF_C	1652	1779	35.31	38.03	4678	BPI_P4	Female	Sugar	Lab	Attica	-
AT_S3_12	1650	3334	33.09	66.87	4986	AT_S3	Adult	Sugar	Wild	Attica	August
TH_S3_46	1645	3309	33.03	66.45	4980	TH_S3	Adult	Sugar	Wild	Thessaly	October
TH_S3_35	1638	3055	32.90	61.36	4979	TH_S3	Adult	Sugar	Wild	Thessaly	October
BPI_P2_1dF_E	1635	1338	36.71	30.04	4454	BPI_P2	Female	Sugar	Lab	Attica	-
BPI_P1_1dF_B	1625	2254	35.86	49.74	4532	BPI_P1	Female	Sugar	Lab	Attica	-
BPI_P4_1dF_E	1611	2869	33.72	60.06	4777	BPI_P4	Female	Sugar	Lab	Attica	-
BPI_P1_14dF_D	1585	1460	32.72	30.14	4844	BPI_P1	Female	Sugar	Lab	Attica	-
BPI_P3_1d_M_B	1580	3191	32.36	65.36	4882	BPI_P3	Male	Sugar	Lab	Attica	-

BPI_P4_3dF_B	1576	1113	32.01	22.60	4924	BPI_P4	Female	Sugar	Lab	Attica	-
BPI_P1_1dF_E	1552	2848	31.36	57.55	4949	BPI_P1	Female	Sugar	Lab	Attica	-
BPI_P4_1dM_E	1540	1393	31.67	28.65	4862	BPI_P4	Male	Sugar	Lab	Attica	-
AT_S3_13	1527	3457	30.60	69.27	4991	AT_S3	Adult	Sugar	Wild	Attica	August
AT_S2_41	1515	3030	31.91	63.82	4748	AT_S2	Adult	Sugar	Wild	Attica	October
UTH_P1_14dF_A	1509	3394	30.45	68.50	4955	UTH_P1	Female	Sugar	Lab	Thessaly	-
TH_S3_33	1493	3428	30.00	68.88	4977	TH_S3	Adult	Sugar	Wild	Thessaly	October
AT_S3_26	1451	3530	29.07	70.73	4991	AT_S3	Adult	Sugar	Wild	Attica	August
AT_S2_19	1450	3136	29.49	63.78	4917	AT_S2	Adult	Sugar	Wild	Attica	October
BPI_P2_3dF_C	1446	995	29.29	20.15	4937	BPI_P2	Female	Sugar	Lab	Attica	-
TH_S1_24	1440	838	29.61	17.23	4863	TH_S1	Adult	Sugar	Wild	Thessaly	June
TH_S2_30	1434	3340	29.49	68.70	4862	TH_S2	Adult	Sugar	Wild	Thessaly	August
BPI_P4_3dF_C	1391	1018	28.19	20.63	4934	BPI_P4	Female	Sugar	Lab	Attica	-
UTH_P2_1D_F_B	1388	3146	28.68	65.00	4840	UTH_P2	Female	Sugar	Lab	Thessaly	-
TH_S2_25	1387	3530	28.08	71.46	4940	TH_S2	Adult	Sugar	Wild	Thessaly	August
UTH_P1_3dF_D	1384	2561	30.42	56.29	4550	UTH_P1	Female	Sugar	Lab	Thessaly	-
UTH_P2_3D_F_B	1384	3051	29.34	64.68	4717	UTH_P2	Female	Sugar	Lab	Thessaly	-
TH_S1_29	1381	1123	46.91	38.15	2944	TH_S1	Adult	Sugar	Wild	Thessaly	June
BPI_P1_14dF_C	1368	1601	27.89	32.64	4905	BPI_P1	Female	Sugar	Lab	Attica	-
BPI_P2_3dF_E	1364	1161	27.51	23.42	4958	BPI_P2	Female	Sugar	Lab	Attica	-
BPI_P4_1dF_B	1326	1623	29.23	35.77	4537	BPI_P4	Female	Sugar	Lab	Attica	-
UTH_P1_3dF_B	1310	3113	27.88	66.25	4699	UTH_P1	Female	Sugar	Lab	Thessaly	-
UTH_P1_1dF_A	1292	1604	28.82	35.78	4483	UTH_P1	Female	Sugar	Lab	Thessaly	-
AT_S3_31	1292	3649	26.08	73.66	4954	AT_S3	Adult	Sugar	Wild	Attica	August
P1_BPI_14dF_C	1284	716	26.43	14.74	4858	BPI_P1_NBF	Female	Sugar	Lab	Attica	-
TH_S2_28	1280	1184	25.96	24.02	4930	TH_S2	Adult	Sugar	Wild	Thessaly	August
BPI_P1_3dF_C	1274	3555	25.89	72.26	4920	BPI_P1	Female	Sugar	Lab	Attica	-
P2_BPI_14dF_A	1257	448	25.35	9.04	4958	BPI_P2_NBF	Female	Sugar	Lab	Attica	-
BPI_P2_1dF_D	1239	1462	27.74	32.74	4466	BPI_P2	Female	Sugar	Lab	Attica	-
AT_S2_29	1226	2217	24.76	44.77	4952	AT_S2	Adult	Sugar	Wild	Attica	October
AT_S3_32	1200	3678	24.38	74.73	4922	AT_S3	Adult	Sugar	Wild	Attica	August
UTH_P1_L3_B	1185	2630	24.43	54.22	4851	UTH_P1	Larva	Sugar	Lab	Thessaly	-
P1_BPI_14dF_B	1161	649	23.48	13.13	4944	BPI_P1_NBF	Female	Sugar	Lab	Attica	-
UTH_P2_L3_C	1157	807	26.45	18.45	4374	UTH_P2	Larva	Sugar	Lab	Thessaly	-
BPI_P4_1dF_D	1149	3243	23.94	67.56	4800	BPI_P4	Female	Sugar	Lab	Attica	-
UTH_P2_L3_E	1126	1644	28.37	41.42	3969	UTH_P2	Larva	Sugar	Lab	Thessaly	-
UTH_P1_L4_A	1115	599	26.12	14.03	4269	UTH_P1	Larva	Sugar	Lab	Thessaly	-

P2_BPI_14dF_D	1115	649	22.57	13.14	4941	BPI_P2_NBF	Female	Sugar	Lab	Attica	-
UTH_P1_1dM_E	1094	1129	24.04	24.81	4550	UTH_P1	Male	Sugar	Lab	Thessaly	-
BPI_P1_14dF_E	1093	919	22.22	18.68	4919	BPI_P1	Female	Sugar	Lab	Attica	-
P1_BPI_14dF_BF_A	1083	744	21.87	15.02	4953	BPI_P1_BF	Female	Blood	Lab	Attica	-
UTH_P2_L3_A	1074	988	24.43	22.47	4397	UTH_P2	Larva	Sugar	Lab	Thessaly	-
AT_S2_17	1066	3862	21.51	77.93	4956	AT_S2	Adult	Sugar	Wild	Attica	October
TH_S1_23	1050	3905	21.06	78.34	4985	TH_S1	Adult	Sugar	Wild	Thessaly	June
BPI_P3_14d_F_C	1043	546	21.36	11.18	4882	BPI_P3	Female	Sugar	Lab	Attica	-
P2_BPI_14dF_C	1033	593	20.79	11.93	4969	BPI_P2_NBF	Female	Sugar	Lab	Attica	-
AT_S3_16	1028	3926	20.69	79.03	4968	AT_S3	Adult	Sugar	Wild	Attica	August
UTH_P1_1dM_D	1025	937	22.23	20.33	4610	UTH_P1	Male	Sugar	Lab	Thessaly	-
UTH_P1_3dM_A	1023	1050	22.50	23.10	4546	UTH_P1	Male	Sugar	Lab	Thessaly	-
P2_BPI_14dF_BF_C	1012	1026	20.67	20.96	4895	BPI_P2_BF	Female	Blood	Lab	Attica	-
TH_S2_8	1010	2877	22.73	64.74	4444	TH_S2	Adult	Sugar	Wild	Thessaly	August
TH_S3_48	1007	3861	20.28	77.75	4966	TH_S3	Adult	Sugar	Wild	Thessaly	October
P1_BPI_14dF_A	995	769	20.50	15.84	4854	BPI_P1_NBF	Female	Sugar	Lab	Attica	-
TH_S2_6	992	2908	21.46	62.92	4622	TH_S2	Adult	Sugar	Wild	Thessaly	August
UTH_P2_1D_M_C	983	1114	24.73	28.03	3975	UTH_P2	Male	Sugar	Lab	Thessaly	-
BPI_P2_3dF_B	981	1603	20.40	33.34	4808	BPI_P2	Female	Sugar	Lab	Attica	-
UTH_P2_1D_M_B	970	3691	20.12	76.58	4820	UTH_P2	Male	Sugar	Lab	Thessaly	-
UTH_P1_L3_A	969	771	21.88	17.41	4428	UTH_P1	Larva	Sugar	Lab	Thessaly	-
UTH_P1_1dM_A	962	1083	21.35	24.04	4505	UTH_P1	Male	Sugar	Lab	Thessaly	-
BPI_P4_3dF_E	941	713	19.20	14.55	4900	BPI_P4	Female	Sugar	Lab	Attica	-
BPI_P4_14dF_C	940	2621	23.13	64.49	4064	BPI_P4	Female	Sugar	Lab	Attica	-
BPI_P3_3d_F_B	932	787	19.46	16.43	4789	BPI_P3	Female	Sugar	Lab	Attica	-
BPI_P3_14d_F_E	932	1102	20.56	24.31	4533	BPI_P3	Female	Sugar	Lab	Attica	-
TH_S2_5	925	1869	20.94	42.31	4417	TH_S2	Adult	Sugar	Wild	Thessaly	August
UTH_P2_3D_M_D	921	3501	19.42	73.83	4742	UTH_P2	Male	Sugar	Lab	Thessaly	-
P2_BPI_14dF_E	919	1285	18.56	25.95	4951	BPI_P2_NBF	Female	Sugar	Lab	Attica	-
TH_S2_20	908	1053	19.93	23.12	4555	TH_S2	Adult	Sugar	Wild	Thessaly	August
BPI_P4_L3_E	901	3534	18.78	73.67	4797	BPI_P4	Larva	Sugar	Lab	Attica	-
UTH_P1_3dM_C	888	1499	18.92	31.94	4693	UTH_P1	Male	Sugar	Lab	Thessaly	-
BPI_P3_14d_F_B	870	521	17.66	10.57	4927	BPI_P3	Female	Sugar	Lab	Attica	-
UTH_P1_L3_C	857	1728	19.44	39.19	4409	UTH_P1	Larva	Sugar	Lab	Thessaly	-
P1_BPI_14dF_D	851	667	17.33	13.58	4911	BPI_P1_NBF	Female	Sugar	Lab	Attica	-
BPI_P2_14dM_C	849	638	21.24	15.96	3997	BPI_P2	Male	Sugar	Lab	Attica	-
P3_BPI_14dF_B	844	930	17.08	18.81	4943	BPI_P3_NBF	Female	Sugar	Lab	Attica	-

TH_S2_4	843	2845	18.17	61.33	4639	TH_S2	Adult	Sugar	Wild	Thessaly	August
BPI_P4_3dF_D	796	483	16.14	9.79	4933	BPI_P4	Female	Sugar	Lab	Attica	-
TH_S3_15	796	2087	16.22	42.51	4909	TH_S3	Adult	Sugar	Wild	Thessaly	October
AT_S2_37	784	1078	16.00	22.00	4901	AT_S2	Adult	Sugar	Wild	Attica	October
AT_S2_11	782	3687	16.00	75.43	4888	AT_S2	Adult	Sugar	Wild	Attica	October
TH_S3_19	766	994	15.37	19.94	4984	TH_S3	Adult	Sugar	Wild	Thessaly	October
UTH_P2_1D_M_E	765	3578	16.35	76.47	4679	UTH_P2	Male	Sugar	Lab	Thessaly	-
UTH_P1_3dM_B	741	1938	15.52	40.58	4776	UTH_P1	Male	Sugar	Lab	Thessaly	-
TH_S2_16	740	3148	14.99	63.75	4938	TH_S2	Adult	Sugar	Wild	Thessaly	August
UTH_P1_3dM_D	717	1450	15.86	32.07	4522	UTH_P1	Male	Sugar	Lab	Thessaly	-
BPI_P2_14dM_D	717	3924	14.74	80.64	4866	BPI_P2	Male	Sugar	Lab	Attica	-
BPI_P3_1d_F_C	709	1312	15.77	29.18	4497	BPI_P3	Female	Sugar	Lab	Attica	-
P2_BPI_14dF_BF_A	706	1674	14.21	33.70	4967	BPI_P2_BF	Female	Blood	Lab	Attica	-
BPI_P4_14dM_B	699	3178	15.45	70.23	4525	BPI_P4	Male	Sugar	Lab	Attica	-
P1_BPI_14dF_BF_B	679	931	13.70	18.79	4955	BPI_P1_BF	Female	Blood	Lab	Attica	-
TH_S2_18	662	4005	13.54	81.90	4890	TH_S2	Adult	Sugar	Wild	Thessaly	August
AT_S2_43	654	4302	13.09	86.09	4997	AT_S2	Adult	Sugar	Wild	Attica	October
TH_S1_38	650	4310	13.05	86.53	4981	TH_S1	Adult	Sugar	Wild	Thessaly	June
P4_BPI_14dF_A	649	1208	13.28	24.72	4886	BPI_P4_NBF	Female	Sugar	Lab	Attica	-
P4_BPI_14dF_BF_E	637	1210	12.88	24.47	4945	BPI_P4_BF	Female	Blood	Lab	Attica	-
UTH_P1_3dM_E	613	1597	12.91	33.62	4750	UTH_P1	Male	Sugar	Lab	Thessaly	-
TH_S3_40	612	4323	12.36	87.28	4953	TH_S3	Adult	Sugar	Wild	Thessaly	October
TH_S3_49	607	4372	12.17	87.69	4986	TH_S3	Adult	Sugar	Wild	Thessaly	October
UTH_P1_L4_C	595	481	14.05	11.36	4234	UTH_P1	Larva	Sugar	Lab	Thessaly	-
UTH_P1_L4_E	593	353	14.71	8.76	4031	UTH_P1	Larva	Sugar	Lab	Thessaly	-
BPI_P3_14d_F_D	580	594	12.01	12.30	4829	BPI_P3	Female	Sugar	Lab	Attica	-
UTH_P2_3D_M_C	576	3870	12.05	80.98	4779	UTH_P2	Male	Sugar	Lab	Thessaly	-
TH_S3_5	570	1463	11.47	29.44	4970	TH_S3	Adult	Sugar	Wild	Thessaly	October
P2_BPI_14dF_BF_B	569	513	11.50	10.37	4948	BPI_P2_BF	Female	Blood	Lab	Attica	-
BPI_P4_14dF_B	566	4157	11.63	85.38	4869	BPI_P4	Female	Sugar	Lab	Attica	-
P1_BPI_14dF_BF_C	558	555	11.30	11.24	4937	BPI_P1_BF	Female	Blood	Lab	Attica	-
AT_S2_45	556	3611	11.12	72.21	5001	AT_S2	Adult	Sugar	Wild	Attica	October
UTH_P1_L4_B	533	387	12.66	9.20	4209	UTH_P1	Larva	Sugar	Lab	Thessaly	-
P4_BPI_14dF_BF_D	533	1126	10.79	22.80	4939	BPI_P4_BF	Female	Blood	Lab	Attica	-
P3_BPI_14dF_BF_B	523	1649	10.56	33.29	4953	BPI_P3_BF	Female	Blood	Lab	Attica	-
UTH_P2_L4_E	512	504	14.55	14.32	3520	UTH_P2	Larva	Sugar	Lab	Thessaly	-
UTH_P2_L4_B	508	678	12.27	16.38	4140	UTH_P2	Larva	Sugar	Lab	Thessaly	-

AT_S2_47	500	2600	10.00	52.00	5000	AT_S2	Adult	Sugar	Wild	Attica	October
UTH_P2_L4_D	495	769	12.97	20.14	3818	UTH_P2	Larva	Sugar	Lab	Thessaly	-
P1_BPI_14dF_BF_D	488	203	9.91	4.12	4925	BPI_P1_BF	Female	Blood	Lab	Attica	-
UTH_P2_L4_C	487	410	13.00	10.95	3745	UTH_P2	Larva	Sugar	Lab	Thessaly	-
BPI_P2_1dM_C	487	428	10.17	8.94	4790	BPI_P2	Male	Sugar	Lab	Attica	-
BPI_P3_1d_M_C	483	4137	10.03	85.88	4817	BPI_P3	Male	Sugar	Lab	Attica	-
P3_BPI_14dF_A	482	912	9.76	18.47	4938	BPI_P3_NBF	Female	Sugar	Lab	Attica	-
P3_BPI_14dF_BF_A	475	733	9.57	14.76	4966	BPI_P3_BF	Female	Blood	Lab	Attica	-
P4_BPI_14dF_BF_B	460	1246	9.27	25.12	4961	BPI_P4_BF	Female	Blood	Lab	Attica	-
P4_BPI_14dF_BF_A	459	710	9.26	14.32	4958	BPI_P4_BF	Female	Blood	Lab	Attica	-
P1_BPI_14dF_BF_E	453	793	9.43	16.50	4806	BPI_P1_BF	Female	Blood	Lab	Attica	-
P3_BPI_14dF_D	448	1182	9.12	24.05	4915	BPI_P3_NBF	Female	Sugar	Lab	Attica	-
TH_S3_2	444	4504	8.96	90.92	4954	TH_S3	Adult	Sugar	Wild	Thessaly	October
BPI_P1_3dM_C	443	1106	11.06	27.62	4005	BPI_P1	Male	Sugar	Lab	Attica	-
P4_BPI_14dF_BF_C	418	1057	8.48	21.43	4932	BPI_P4_BF	Female	Blood	Lab	Attica	-
UTH_P1_1dM_C	416	1713	11.01	45.32	3780	UTH_P1	Male	Sugar	Lab	Thessaly	-
P4_BPI_14dF_B	413	1335	8.40	27.15	4917	BPI_P4_NBF	Female	Sugar	Lab	Attica	-
BPI_P2_1dM_E	411	460	8.81	9.86	4666	BPI_P2	Male	Sugar	Lab	Attica	-
BPI_P2_14dF_E	408	1429	9.52	33.34	4286	BPI_P2	Female	Sugar	Lab	Attica	-
P3_BPI_14dF_BF_E	400	557	8.08	11.25	4951	BPI_P3_BF	Female	Blood	Lab	Attica	-
P1_BPI_14dF_E	394	295	8.03	6.01	4908	BPI_P1_NBF	Female	Sugar	Lab	Attica	-
TH_S2_29	390	3874	7.99	79.34	4883	TH_S2	Adult	Sugar	Wild	Thessaly	August
TH_S2_27	371	4575	7.48	92.26	4959	TH_S2	Adult	Sugar	Wild	Thessaly	August
UTH_P2_3D_M_A	368	3787	7.78	80.03	4732	UTH_P2	Male	Sugar	Lab	Thessaly	-
UTH_P2_14D_M_B	364	3684	7.45	75.45	4883	UTH_P2	Male	Sugar	Lab	Thessaly	-
BPI_P2_3dM_C	362	781	7.81	16.84	4638	BPI_P2	Male	Sugar	Lab	Attica	-
P4_BPI_14dF_D	352	297	7.12	6.01	4942	BPI_P4_NBF	Female	Sugar	Lab	Attica	-
UTH_P2_L4_A	352	434	9.47	11.68	3716	UTH_P2	Larva	Sugar	Lab	Thessaly	-
TH_S3_14	341	4613	6.83	92.43	4991	TH_S3	Adult	Sugar	Wild	Thessaly	October
BPI_P2_14dF_C	328	1066	7.84	25.49	4182	BPI_P2	Female	Sugar	Lab	Attica	-
UTH_P1_L4_D	320	503	7.85	12.33	4078	UTH_P1	Larva	Sugar	Lab	Thessaly	-
AT_S1_17	317	232	6.41	4.69	4945	AT_S1	Adult	Sugar	Wild	Attica	June
BPI_P2_1dM_D	312	773	6.78	16.81	4599	BPI_P2	Male	Sugar	Lab	Attica	-
BPI_P3_1d_F_E	303	951	8.74	27.43	3467	BPI_P3	Female	Sugar	Lab	Attica	-
BPI_P4_3dM_D	293	257	6.03	5.29	4858	BPI_P4	Male	Sugar	Lab	Attica	-
P3_BPI_14dF_BF_D	285	224	5.74	4.51	4962	BPI_P3_BF	Female	Blood	Lab	Attica	-
BPI_P2_3dM_E	280	746	5.96	15.87	4702	BPI_P2	Male	Sugar	Lab	Attica	-

P2_BPI_14dF_B	279	368	5.64	7.44	4948	BPI_P2_NBF	Female	Sugar	Lab	Attica	-
P3_BPI_14dF_E	279	1611	5.65	32.62	4939	BPI_P3_NBF	Female	Sugar	Lab	Attica	-
BPI_P3_3d_F_E	278	1432	6.11	31.48	4549	BPI_P3	Female	Sugar	Lab	Attica	-
BPI_P2_1dM_B	277	456	5.72	9.41	4847	BPI_P2	Male	Sugar	Lab	Attica	-
UTH_P2_1D_M_D	277	4172	5.82	87.67	4759	UTH_P2	Male	Sugar	Lab	Thessaly	-
TH_S3_1	274	4705	5.49	94.27	4991	TH_S3	Adult	Sugar	Wild	Thessaly	October
BPI_P4_L3_B	268	4019	5.66	84.91	4733	BPI_P4	Larva	Sugar	Lab	Attica	-
P3_BPI_14dF_BF_C	266	681	5.37	13.75	4953	BPI_P3_BF	Female	Blood	Lab	Attica	-
TH_S2 _7	262	3157	6.30	75.89	4160	TH_S2	Adult	Sugar	Wild	Thessaly	August
TH_S1_36	242	2742	5.26	59.62	4599	TH_S1	Adult	Sugar	Wild	Thessaly	June
P4_BPI_14dF_E	239	786	4.84	15.92	4936	BPI_P4_NBF	Female	Sugar	Lab	Attica	-
BPI_P3_3d_M_C	237	4223	5.05	90.04	4690	BPI_P3	Male	Sugar	Lab	Attica	-
BPI_P2_1dF_C	235	591	6.11	15.38	3844	BPI_P2	Female	Sugar	Lab	Attica	-
BPI_P2_14dF_D	229	1601	6.20	43.36	3692	BPI_P2	Female	Sugar	Lab	Attica	-
UTH_P2_14D_M_A	225	3754	4.74	79.12	4745	UTH_P2	Male	Sugar	Lab	Thessaly	-
AT_S1_46	213	3654	4.46	76.56	4773	AT_S1	Adult	Sugar	Wild	Attica	June
AT_S2_35	212	3811	4.35	78.27	4869	AT_S2	Adult	Sugar	Wild	Attica	October
BPI_P2_L3_E	207	810	4.29	16.79	4825	BPI_P2	Larva	Sugar	Lab	Attica	-
BPI_P3_3d_M_D	205	3459	4.48	75.56	4578	BPI_P3	Male	Sugar	Lab	Attica	-
TH_S3_36	190	982	3.81	19.71	4983	TH_S3	Adult	Sugar	Wild	Thessaly	October
BPI_P3_1d_M_E	190	3607	4.25	80.66	4472	BPI_P3	Male	Sugar	Lab	Attica	-
P3_BPI_14dF_C	188	351	3.79	7.08	4961	BPI_P3_NBF	Female	Sugar	Lab	Attica	-
BPI_P4_3dM_E	187	178	3.88	3.69	4818	BPI_P4	Male	Sugar	Lab	Attica	-
UTH_P1_1dM_B	185	498	7.14	19.21	2592	UTH_P1	Male	Sugar	Lab	Thessaly	-
TH_S3_47	183	354	3.70	7.15	4948	TH_S3	Adult	Sugar	Wild	Thessaly	October
UTH_P2_3D_M_B	179	3784	3.98	84.13	4498	UTH_P2	Male	Sugar	Lab	Thessaly	-
BPI_P3_3d_M_B	174	2964	3.90	66.43	4462	BPI_P3	Male	Sugar	Lab	Attica	-
BPI_P2_3dM_D	164	387	3.51	8.28	4674	BPI_P2	Male	Sugar	Lab	Attica	-
BPI_P4_3dM_C	160	187	3.34	3.90	4798	BPI_P4	Male	Sugar	Lab	Attica	-
UTH_P2_3D_M_E	159	3927	3.47	85.59	4588	UTH_P2	Male	Sugar	Lab	Thessaly	-
BPI_P1_L4_C	154	318	3.23	6.67	4770	BPI_P1	Larva	Sugar	Lab	Attica	-
TH_S3_34	150	708	3.01	14.22	4978	TH_S3	Adult	Sugar	Wild	Thessaly	October
BPI_P3_1d_F_D	144	1748	3.92	47.57	3675	BPI_P3	Female	Sugar	Lab	Attica	-
P4_BPI_14dF_C	143	480	2.90	9.72	4936	BPI_P4_NBF	Female	Sugar	Lab	Attica	-
BPI_P3_L3_E	138	1114	3.04	24.52	4543	BPI_P3	Larva	Sugar	Lab	Attica	-
BPI_P4_14dF_D	138	2708	3.30	64.75	4182	BPI_P4	Female	Sugar	Lab	Attica	-
BPI_P1_3dM_D	136	1948	3.41	48.88	3985	BPI_P1	Male	Sugar	Lab	Attica	-

BPI_P1_L4_E	133	146	2.71	2.98	4906	BPI_P1	Larva	Sugar	Lab	Attica	-
BPI_P1_1dM_E	132	2063	4.61	72.06	2863	BPI_P1	Male	Sugar	Lab	Attica	-
BPI_P1_3dM_E	132	2607	3.16	62.43	4176	BPI_P1	Male	Sugar	Lab	Attica	-
BPI_P4_L3_D	129	4596	2.65	94.41	4868	BPI_P4	Larva	Sugar	Lab	Attica	-
BPI_P2_14dF_B	128	1026	3.03	24.25	4231	BPI_P2	Female	Sugar	Lab	Attica	-
BPI_P4_L3_C	127	3272	2.63	67.67	4835	BPI_P4	Larva	Sugar	Lab	Attica	-
AT_S2_13	127	4671	2.59	95.35	4899	AT_S2	Adult	Sugar	Wild	Attica	October
BPI_P3I_L4_B	124	223	2.59	4.65	4797	BPI_P3	Larva	Sugar	Lab	Attica	-
BPI_P1_L3_C	117	2971	2.46	62.34	4766	BPI_P1	Larva	Sugar	Lab	Attica	-
AT_S1_19	112	131	2.70	3.16	4152	AT_S1	Adult	Sugar	Wild	Attica	June
BPI_P3_L4_C	112	165	2.59	3.81	4328	BPI_P3	Larva	Sugar	Lab	Attica	-
UTH_P2_14D_M_C	107	3841	2.31	82.75	4642	UTH_P2	Male	Sugar	Lab	Thessaly	-
BPI_P4_1dM_D	99	215	2.04	4.43	4850	BPI_P4	Male	Sugar	Lab	Attica	-
BPI_P3_3d_F_D	97	3369	2.25	78.04	4317	BPI_P3	Female	Sugar	Lab	Attica	-
BPI_P2_3dF_D	95	65	2.10	1.44	4526	BPI_P2	Female	Sugar	Lab	Attica	-
BPI_P4_1dM_C	88	147	1.80	3.01	4880	BPI_P4	Male	Sugar	Lab	Attica	-
BPI_P1_1dM_B	87	4588	1.77	93.10	4928	BPI_P1	Male	Sugar	Lab	Attica	-
BPI_P2_L4_D	84	230	1.71	4.67	4927	BPI_P2	Larva	Sugar	Lab	Attica	-
UTH_P1_14dM_E	76	4564	1.56	93.39	4887	UTH_P1	Male	Sugar	Lab	Thessaly	-
BPI_P1_L4_B	72	260	1.48	5.34	4873	BPI_P1	Larva	Sugar	Lab	Attica	-
BPI_P2_L3_B	71	450	1.46	9.28	4850	BPI_P2	Larva	Sugar	Lab	Attica	-
TH_S2 _9	70	379	1.49	8.05	4711	TH_S2	Adult	Sugar	Wild	Thessaly	August
BPI_P2_3dM_B	70	673	1.50	14.37	4683	BPI_P2	Male	Sugar	Lab	Attica	-
BPI_P4_3dM_B	66	129	1.39	2.71	4762	BPI_P4	Male	Sugar	Lab	Attica	-
BPI_P2_L3_D	65	968	1.56	23.26	4161	BPI_P2	Larva	Sugar	Lab	Attica	-
BPI_P3_L4_E	59	910	1.25	19.31	4713	BPI_P3	Larva	Sugar	Lab	Attica	-
UTH_P1_14dM_A	58	4871	1.17	98.19	4961	UTH_P1	Male	Sugar	Lab	Thessaly	-
BPI_P1_1dF_D	52	1856	1.12	39.98	4642	BPI_P1	Female	Sugar	Lab	Attica	-
BPI_P3_L4_D	50	775	1.12	17.36	4465	BPI_P3	Larva	Sugar	Lab	Attica	-
TH_S1_31	48	4725	0.97	95.82	4931	TH_S1	Adult	Sugar	Wild	Thessaly	June
BPI_P1_L3_D	46	955	0.95	19.76	4833	BPI_P1	Larva	Sugar	Lab	Attica	-
AT_S3_28	44	4926	0.88	98.82	4985	AT_S3	Adult	Sugar	Wild	Attica	August
BPI_P3_L3_C	43	190	0.89	3.92	4844	BPI_P3	Larva	Sugar	Lab	Attica	-
BPI_P1_1dM_D	42	1988	1.03	48.76	4077	BPI_P1	Male	Sugar	Lab	Attica	-
AT_S1_48	41	2485	0.84	50.94	4878	AT_S1	Adult	Sugar	Wild	Attica	June
UTH_P2_14D_M_D	41	4552	0.85	94.32	4826	UTH_P2	Male	Sugar	Lab	Thessaly	-
BPI_P4_1dM_B	38	40	0.77	0.81	4935	BPI_P4	Male	Sugar	Lab	Attica	-

BPI_P3_L3_D	38	255	0.83	5.55	4596	BPI_P3	Larva	Sugar	Lab	Attica	-
BPI_P1_L4_D	37	301	0.85	6.88	4375	BPI_P1	Larva	Sugar	Lab	Attica	-
BPI_P3_1d_M_D	35	2997	0.82	69.83	4292	BPI_P3	Male	Sugar	Lab	Attica	-
BPI_P3_3d_M_E	31	2181	0.79	55.27	3946	BPI_P3	Male	Sugar	Lab	Attica	-
BPI_P1_L3_E	29	173	0.60	3.59	4817	BPI_P1	Larva	Sugar	Lab	Attica	-
BPI_P2_L3_C	27	813	0.56	16.91	4807	BPI_P2	Larva	Sugar	Lab	Attica	-
UTH_P1_14dM_C	26	4827	0.53	98.01	4925	UTH_P1	Male	Sugar	Lab	Thessaly	-
TH_S1_20	26	4926	0.52	99.16	4968	TH_S1	Adult	Sugar	Wild	Thessaly	June
BPI_P3_L3_B	23	216	0.47	4.46	4848	BPI_P3	Larva	Sugar	Lab	Attica	-
BPI_P4_L4_B	23	1166	0.47	23.65	4931	BPI_P4	Larva	Sugar	Lab	Attica	-
BPI_P1_14dM_C	20	601	0.42	12.54	4791	BPI_P1	Male	Sugar	Lab	Attica	-
BPI_P1_1dM_C	20	1604	0.54	42.99	3731	BPI_P1	Male	Sugar	Lab	Attica	-
AT_S3_37	19	3747	0.39	76.30	4911	AT_S3	Adult	Sugar	Wild	Attica	August
BPI_P4_L4_D	16	1311	0.33	26.69	4912	BPI_P4	Larva	Sugar	Lab	Attica	-
BPI_P3_14d_M_D	13	855	0.27	17.47	4894	BPI_P3	Male	Sugar	Lab	Attica	-
AT_S1_43	13	1560	0.35	41.94	3720	AT_S1	Adult	Sugar	Wild	Attica	June
UTH_P2_14D_M_E	13	4429	0.27	90.81	4877	UTH_P2	Male	Sugar	Lab	Thessaly	-
UTH_P1_14dM_B	13	4737	0.27	97.35	4866	UTH_P1	Male	Sugar	Lab	Thessaly	-
BPI_P2_L4_E	11	1253	0.22	25.25	4962	BPI_P2	Larva	Sugar	Lab	Attica	-
BPI_P1_14dM_B	10	569	0.21	11.79	4826	BPI_P1	Male	Sugar	Lab	Attica	-
AT_S3_30	10	4952	0.20	99.58	4973	AT_S3	Adult	Sugar	Wild	Attica	August
AT_S1_42	7	866	0.18	22.10	3919	AT_S1	Adult	Sugar	Wild	Attica	June
BPI_P3_14d_M_E	7	2727	0.16	63.95	4264	BPI_P3	Male	Sugar	Lab	Attica	-
BPI_P4_L4_E	6	299	0.12	6.07	4927	BPI_P4	Larva	Sugar	Lab	Attica	-
BPI_P3_14d_M_B	5	233	0.10	4.73	4927	BPI_P3	Male	Sugar	Lab	Attica	-
BPI_P2_L4_C	5	272	0.10	5.50	4948	BPI_P2	Larva	Sugar	Lab	Attica	-
UTH_P1_14dM_D	5	4879	0.10	98.65	4946	UTH_P1	Male	Sugar	Lab	Thessaly	-
TH_S1_18	5	4927	0.10	99.52	4951	TH_S1	Adult	Sugar	Wild	Thessaly	June
TH_S1_25	3	35	0.06	0.74	4752	TH_S1	Adult	Sugar	Wild	Thessaly	June
BPI_P1_14dM_E	3	147	0.06	3.08	4768	BPI_P1	Male	Sugar	Lab	Attica	-
BPI_P4_L4_C	2	530	0.04	10.71	4948	BPI_P4	Larva	Sugar	Lab	Attica	-
BPI_P3_14d_M_C	2	1314	0.04	26.79	4905	BPI_P3	Male	Sugar	Lab	Attica	-
AT_S1_50	2	4518	0.04	99.41	4545	AT_S1	Adult	Sugar	Wild	Attica	June
BPI_P1_14dM_D	1	1014	0.02	20.88	4857	BPI_P1	Male	Sugar	Lab	Attica	-
BPI_P4_14dF_E	0	2708	0.00	64.45	4202	BPI_P4	Female	Sugar	Lab	Attica	-
**Total**	**374074 reads**	**729231 reads**	**-**	**-**	**1716477 reads**						
**Average**	**1031 reads**	**2009 reads**	**21.50%**	**42.23%**							

Table S9. The core bacterial species based on the origin of the population. Presence in a sample group is denoted with "1" and absence with "0".

OTU ID	Phylum	Class	Order	Family	Genus	Species	Attica Lab	Attica Wild	Thessaly Lab	Thessaly Wild
OTU1	Proteobacteria	Alphaproteobacteria	Rickettsiales	Anaplasmataceae	*Wolbachia*	*Wolbachia* sp.	1	1	1	1
OTU10	Proteobacteria	Gammaproteobacteria	Pseudomonadales	Pseudomonadaceae	*Pseudomonas*	*Pseudomonas* sp.	1	1	1	1
OTU12	Proteobacteria	Alphaproteobacteria	Rhizobiales	Rhizobiaceae	*Phyllobacterium*	*Phyllobacterium* sp.	0	0	1	0
OTU2	Bacteroidota	Bacteroidia	Flavobacteriales	Weeksellaceae	*Elizabethkingia*	*Elizabethkingia ursingii*	1	0	1	1
OTU28	Actinobacteriota	Actinobacteria	Propionibacteriales	Propionibacteriaceae	*Cutibacterium*	*Cutibacterium* sp.	0	0	1	1
OTU3	Proteobacteria	Alphaproteobacteria	Rickettsiales	Anaplasmataceae	*Wolbachia*	*Wolbachia* of *Calligrapha confluens*	1	1	1	1
OTU4	Proteobacteria	Gammaproteobacteria	Enterobacterales	Yersiniaceae	*Serratia*	*Serratia marcescens*	1	0	1	0
OTU42	Proteobacteria	Gammaproteobacteria	Vibrionales	Vibrionaceae	*Vibrio*	*Vibrio metschnikovii*	0	0	1	0
OTU5	Proteobacteria	Alphaproteobacteria	Acetobacterales	Acetobacteraceae	*Asaia*	*Asaia siamensis*	1	0	1	0

Table S10. The core bacteria of the laboratory populations based on the developmental stage. Presence is denoted with "1" and absence with "0".

OUT ID	Phylum	Class	Order	Family	Genus	Species	BPI - adult	BPI - larva	UTH - adult	UTH - larva
Otu1	Proteobacteria	Alphaproteobacteria	Rickettsiales	Anaplasmataceae	*Wolbachia*	*Wolbachia* sp.	1	1	1	1
Otu10	Proteobacteria	Gammaproteobacteria	Pseudomonadales	Pseudomonadaceae	*Pseudomonas*	*Pseudomonas* sp.	1	1	1	1
Otu11	Proteobacteria	Alphaproteobacteria	Rhizobiales	Beijerinckiaceae	*Bosea*	*Bosea* sp.	0	0	0	1
Otu12	Proteobacteria	Alphaproteobacteria	Rhizobiales	Rhizobiaceae	*Phyllobacterium*	*Phyllobacterium* sp.	0	0	1	0
Otu13	Proteobacteria	Gammaproteobacteria	Pseudomonadales	Moraxellaceae	*Acinetobacter*	*Acinetobacter johnsonii*	0	1	0	1
Otu2	Bacteroidota	Bacteroidia	Flavobacteriales	Weeksellaceae	*Elizabethkingia*	*Elizabethkingia ursingii*	1	1	1	1
Otu22	Proteobacteria	Alphaproteobacteria	Sphingomonadales	Sphingomonadaceae	*Sphingomonas*	*Sphingomonas* sp.	0	0	0	1
Otu23	Proteobacteria	Alphaproteobacteria	Sphingomonadales	Sphingomonadaceae	*Sphingobium*	*Sphingobium* sp.2	0	0	0	1
Otu28	Actinobacteriota	Actinobacteria	Propionibacteriales	Propionibacteriaceae	*Cutibacterium*	*Cutibacterium* sp.	0	0	1	1
Otu3	Proteobacteria	Alphaproteobacteria	Rickettsiales	Anaplasmataceae	*Wolbachia*	*Wolbachia* of *Calligrapha confluens*	1	1	1	1
Otu30	Proteobacteria	Gammaproteobacteria	Aeromonadales	Aeromonadaceae	*Aeromonas*	*Aeromonas* sp.	0	0	1	0
Otu31	Proteobacteria	Alphaproteobacteria	Caulobacterales	Caulobacteraceae	*Brevundimonas*	*Brevundimonas vesicularis*	0	0	0	1
Otu36	Proteobacteria	Alphaproteobacteria	Rhizobiales	Rhizobiaceae	*Allorhizobium-Neorhizobium-Pararhizobium-Rhizobium*	*Rhizobium* sp.	0	0	0	1
Otu38	Proteobacteria	Gammaproteobacteria	Burkholderiales	Comamonadaceae	*Delftia*	*Delftia acidovorans*	0	0	0	1

Otu4	Proteobacteria	Gammaproteobacteria	Enterobacterales	Yersiniaceae	*Serratia*	*Serratia marcescens*	1	0	1	1
Otu42	Proteobacteria	Gammaproteobacteria	Vibrionales	Vibrionaceae	*Vibrio*	*Vibrio metschnikovii*	0	0	1	1
Otu45	Proteobacteria	Gammaproteobacteria	Pseudomonadales	Pseudomonadaceae	*Pseudomonas*	*Pseudomonas resinovorans*	0	0	0	1
Otu47	Proteobacteria	Alphaproteobacteria	Sphingomonadales	Sphingomonadaceae	*Sphingomonas*	*Sphingomonas koreensis*	0	0	0	1
Otu48	Proteobacteria	Gammaproteobacteria	Pseudomonadales	Moraxellaceae	*Enhydrobacter*	*Enhydrobacter* sp.	0	0	0	1
Otu5	Proteobacteria	Alphaproteobacteria	Acetobacterales	Acetobacteraceae	*Asaia*	*Asaia siamensis*	1	0	1	1

Table S11. Isolated bacterial strains from larval and adult extracts grown on three culturing media.

Developmental stage	Culturing media	Morphologically unique cultures
**Larval extract**	1. LB Agar	3
	2. PDA	7
	3. MacConkey Agar	7
	**Total**	**17**
**Adult extract**	1. LB Agar	20
	2. PDA	8
	3. MacConkey Agar	7
	**Total**	**35**

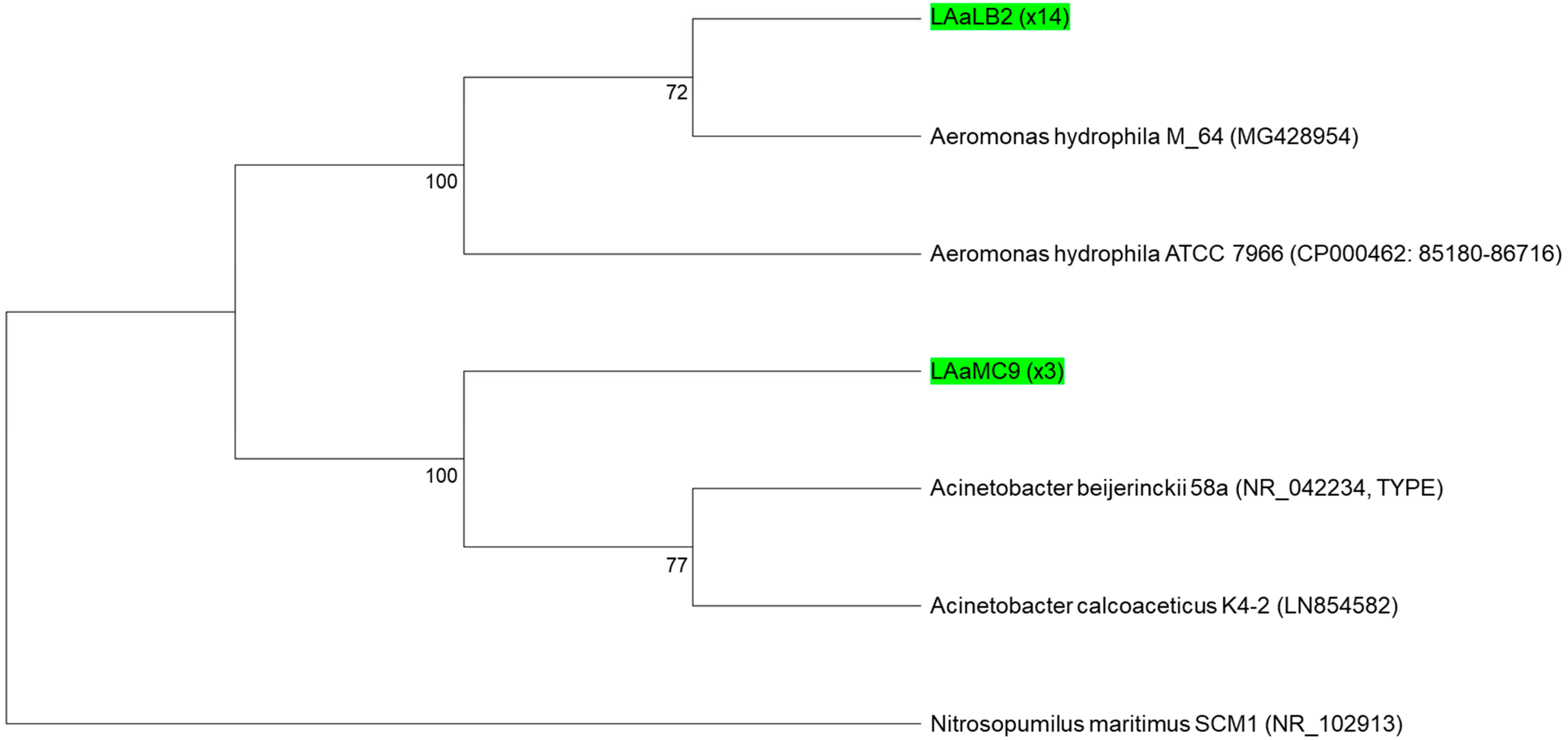

Figure S12. Neighbor-joining tree of 16S rRNA sequences of bacterial strains isolated from the larval extract. The size of the fragment used for the tree was ~825 bp. Accession numbers of reference sequences are included in parentheses. Isolated strains are highlighted in green (parentheses denote the number of identical sequences).
